# The Holozoan *Capsaspora owczarzaki* Possesses a Diverse Complement of Active Transposable Element Families

**DOI:** 10.1093/gbe/evu068

**Published:** 2014-04-02

**Authors:** Martin Carr, Hiroshi Suga

**Affiliations:** ^1^School of Applied Sciences, University of Huddersfield, West Yorkshire, United Kingdom; ^2^Department of Life Sciences Faculty of Life and Environmental Science, Hiroshima Prefectural University, Hiroshima, Japan

**Keywords:** Filasterea, phylogenetics, population genetics, retrotransposons, DNA transposons

## Abstract

*Capsaspora owczarzaki*, a protistan symbiont of the pulmonate snail *Biomphalaria glabrata*, is the centre of much interest in evolutionary biology due to its close relationship to Metazoa. The whole genome sequence of this protist has revealed new insights into the ancestral genome composition of Metazoa, in particular with regard to gene families involved in the evolution of multicellularity. The draft genome revealed the presence of 23 families of transposable element, made up from DNA transposon as well as long terminal repeat (LTR) and non-LTR retrotransposon families. The phylogenetic analyses presented here show that all of the transposable elements identified in the *C. owczarzaki* genome have orthologous families in Metazoa, indicating that the ancestral metazoan also had a rich diversity of elements. Molecular evolutionary analyses also show that the majority of families has recently been active within the *Capsaspora* genome. One family now appears to be inactive and a further five families show no evidence of current transposition. Most individual element copies are evolutionarily young; however, a small proportion of inserts appear to have persisted for longer in the genome. The families present in the genome show contrasting population histories and appear to be in different stages of their life cycles. Transcriptome data have been analyzed from multiple stages in the *C. owczarzaki* life cycle. Expression levels vary greatly both between families and between different stages of the life cycle, suggesting an unexpectedly complex level of transposable element regulation in a single celled organism.

## Introduction

The eukaryotic supergroup Opisthokonta contains, in the Metazoa and Fungi, two of the major multicellular eukaryotic lineages. In addition to the multicellular groups, however, the opisthokonts also comprise the protistan groups Choanoflagellatea, Corallochytrea, Filasterea, Ichthyosporea, and the nuclearioid amoebae (Carr and Baldauf 2011). The evolutionary relationships within the opisthokonts are slowly becoming clear, with the group being divided into two major lineages. The Holomycota (Liu et al. 2009) is composed of Fungi and their sister group the nuclearioid amoebae, whereas the remaining opisthokont groups make up the Holozoa (Lang et al. 2002; Shalchian-Tabrizi et al. 2008).

*Capsaspora owczarzaki* is, along with two species of *Ministeria*, one of the three protist taxa assigned to Filasterea (Shalchian-Tabrizi et al. 2008) and is the sole known representative of the genus *Capsaspora*. The species is of biological interest for two important reasons. First, *C*. *owczarzaki* has a symbiotic, or parasitic, relationship with the snail *Biomphalaria glabrata*, which acts as a vector in the transmission of the human disease schistosomiasis (Sohn and Kornicker 1972). This chronic disease is estimated by the World Health Organization to affect over 240 million people and annually results in over 200,000 deaths (WHO Fact sheet No. 115, 2013). Schistosomiasis is caused by the trematode worm *Schistosoma mansoni*, which uses *B. glabrata* as an intermediate host. The presence of *C. owczarzaki* may afford *B. glabrata* resistance to *Schistosoma* infection as it preys upon the trematode larvae in the snail haemolymph (Stibbs et al. 1979; Owczarzak et al. 1980), which in turn has led to speculation that *C. owczarzaki* has a potential role as a biological control of schistosomiasis (Ruiz-Trillo et al. 2006).

Second, *C. owczarzaki* is a close relative of the metazoans and the choanoflagellates (Shalchian-Tabrizi et al. 2008; Torruella et al. 2012) and is therefore an important study taxon in the transition from the unicellular protists to Metazoa (Ruiz-Trillo, Roger et al. 2008; Sebé-Pedrós et al. 2011; Suga et al. 2013). The genome of *C. owczarzaki*, sequenced at the Broad Institute, Massachusetts under the Origins of Multicellularity Initiative (Suga et al. 2013), is 27.97 Mb in length and contains over 8,000 predicted genes. The genome size is therefore similar to many fungal taxa and unicellular eukaryotes although smaller than most metazoan genomes (Carr and Baldauf 2011).

Transposable elements (TEs) are important components of eukaryotic genomes, acting as a source of deleterious mutations, genetic variability, phylogenetic markers, and beneficial domestication by the host, as well as being a component of genomic architecture (Eanes et al. 1988; Capy et al. 2000; Carr et al. 2001; Jordan et al. 2003; Casola et al. 2007; Biémont 2009). TEs fall into two classes, defined by their mode of transposition. Class I elements are retrotransposons, either with or without long terminal repeats (LTRs), which transpose via an RNA intermediate. LTR elements are commonly found in their host genomes in two different forms. Full length elements (FLE) are composed of two LTRs that flank *gag* and *pol* open reading frames (ORFs), which encode structural and replication proteins, respectively. The second form of LTR retrotransposon is the solo LTR. The two LTR sequences of a single element are capable of undergoing ectopic recombination with each other. This process leads to the excision of one LTR and the internal DNA, as an extrachromosomal circular element, and leaves a single LTR present in host chromosome. Transposition of all retrotransposons is facilitated by a polyprotein (Pol), a multifunction protein which encodes reverse transcriptase and integrase domains. Non-LTR retrotransposons lack terminal repeats; however, like the LTR elements, they contain both *gag* and *pol* ORFs and transpose via an RNA intermediate. Daughter non-LTR elements are frequently “dead on arrival,” due to the propensity of non-LTR retrotransposons to undergo 5′ truncations during transposition (Malik and Eickbush 1998).

Class II elements, the DNA transposons, exist solely as DNA. They usually employ a simple “cut and paste mechanism” of transposition via their transposase (Tnpase) protein, in which the entire element is excised from the host chromosome and inserted into a new genomic location.

TEs have traditionally been considered as either selfish or junk DNA conferring no benefit to their host. This view has been challenged with clear examples of both beneficial individual insertions (Franchini et al. 2004; Schlenke and Begun 2004) and TE families (Biessmann et al. 1992). The idea that TEs are solely genomic parasites now appears overly simplistic; however, the majority of insertions appears to be deleterious or neutral in a broad range of eukaryotes (Charlesworth et al. 1992; Jordan and McDonald 1999; Pereira 2004). Their deleterious nature results in natural selection playing an important role in restraining the proliferation of TEs in many host populations.

Most studies on TEs in eukaryotes have centered on the major multicellular groups, that is, Metazoa, Fungi, and plants, with relatively little known on the function and evolution of TEs in protists. To date only a single choanoflagellate, which are the sister group to metazoans, has been studied within the opisthokont protists with regard to the evolution of their TEs (Carr et al. 2008). *Monosiga brevicollis* was shown to harbor three TE families, all of which were LTR retrotransposons and active. Carr et al. (2008) also showed that TEs only constitute a very low fraction (∼1%) of the *M. brevicollis* genome, with families in a state of constant turnover through ongoing transposition and loss possibly due to natural selection.

The *C. owczarzaki* genome allows further insight into the evolution of TEs in the opisthokont protists. The recently published *C. owczarzaki* draft genome identified 23 TE families, comprising five LTR retrotransposon, four non-LTR retrotransposon, and 14 DNA transposon families. The 23 families were shown to belong to seven major superfamilies of TE and contributed to approximately 9% of the genome (Suga et al. 2013).

Presented here is a detailed characterization of the TEs present in the draft *C. owczarzaki* genome with an emphasis on their evolutionary biology. All of the families have been placed in a phylogenetic framework and are shown to cluster together to the exclusion of non-*Capsaspora* families. The *C. owczarzaki* families are generally isolated on long internal branches; however, they do form phylogenetic associations with TEs from other opisthokont taxa. The data presented here give an insight into the ancestral TE composition of Metazoa as all of the families identified in the *C. owczarzaki* genome have orthologous families in metazoan taxa. The seven superfamilies present in *C. owczarzaki* appear to have been present in the last common ancestor of metazoans and filastereans and subsequently retained in both lineages.

Through molecular evolutionary and phylogenetic analyses of the individual element copies, it can be seen that 22 of the 23 families have recently undergone transposition within the sequenced genome; however, one of the families has subsequently lost the ability to transpose. The TE population of *C. owczarzaki* is dominated by young elements; however, the presence of ancient inserts, as well as divergent subfamilies, highlights that many families are long-term components of the genome. The families show contrasting population histories within *C. owczarzaki*; two families appear to be possible recent arrivals in the genome and five of the families show no evidence of current transposition.

Finally, the expression levels of each family have been determined in three different stages of the *C. owczarzaki* life cycle. The data suggest a strong relationship between the rates of expression and transposition in *Capsaspora* and, unexpectedly for a single celled organism, stage-specific expression for families.

## Materials and Methods

### Extraction of Individual Element Termini and Transcripts

The termini of individual copies were extracted by Megablast similarity searches, using default parameters, on the *C. owczarzaki* Trace Archive using the reported sequences of each family, taken from the draft genome paper (Suga et al. 2013), as query sequences. The query sequences for the LTR retrotransposon families were the LTR sequences; 5′ and 3′ noncoding regions were used as queries for the non-LTR retrotransposons and the DNA transposon families. Query sequences were limited to a maximum of 300 bp. The integration of a TE generally results in the duplication of the target site, with the same sequence being present at both the 5′ and 3′ termini. The termini for individual inserts were identified through the flanking DNA and target site duplications (TSDs) generated upon integration. The presence of both TSDs in the Trace Archive allowed the two ends of individual inserts to be identified.

Transcriptome data were generated from three stages of the *C. owczarzaki* life cycle (Sebé-Pedrós et al. 2013), with three replicates produced for each stage, and are available in the NCBI BioProject PRJNA20341. The raw reads from the transcriptome were mapped to the full length sequences of each family using SMALT (Hannes Ponstingl, SMALT—Wellcome Trust Sanger Institute, http://www.sanger.ac.uk/resources/software/smalt/, last accessed April 12, 2014). The expressions of TEs were approximated by the normalized number of RNAseq reads and visualized with a heatmap. The values were normalized by the maximum value (i.e., *Cocv1* in the adherent stage) and the heatmap was drawn in R 3.0.2 using its Bioconductor package (Gentleman et al. 2004). As the expression levels of some families were extremely high, the same heatmap was also drawn by log scale, in which the minimum value is set to zero. Differences in expression level between stages in the life cycle were tested using analysis of variance for each family. For those families which showed significant variation across the three stages, the data were examined to see whether one stage showed significantly elevated expression. To this end, for each family, the three replicates from the two stages with the highest combined expression level were compared using an unpaired *t*-test.

### Phylogenetic and Molecular Evolution Analyses

Superfamily phylogenies were created for all of the families in the *C. owczarzaki* genome using the amino acid sequences of Pol, in the case of retrotransposons, and Tnpase in the case of DNA transposons. In order to identify families closely related to the *C. owczarzaki* families, sequence similarity searches were performed with BLASTp and tBLASTn on the National Center for Biotechnology Information (NCBI) Nonredundant Proteins Sequences and Nucleotide Collection databases, respectively, using default parameters. Further searches were performed on the whole genome sequences of a taxonomically broad set of eukaryotes following the protocol set out in Suga et al. (2013) (Amoebozoa: *Dictyostelium discoideum*; Apusozoa: *Thecamonas trahens*; Choanoflagellatea: *M. brevicollis*, *Salpingoeca rosetta*; Excavata: *Naegleria gruberi*; Fungi: *Laccaria bicolor*, *Rhizopus delemar*; Metazoa: *Amphimedon queenslandica*, *Drosophila melanogaster, Homo sapiens*, *Nematostella vectensis*, *Trichoplax adhaerens*) with both BLASTp and tBLASTn.

Amino acid sequences were aligned using MUSCLE 3.7 (Edgar 2004), on the EMBL-EBI server using default parameters, and modified by eye to minimize indel regions. In order to determine the appropriate amino acid substitution model for the maximum likelihood phylogenetic analyses, the alignments were analyzed with ProtTest 2.4 (Abascal et al. 2005). Maximum likelihood analyses were performed using RAxML 7.2.6 (Stamatakis et al. 2005) on raxmlGUI2 (Silvestro 2012). The maximum likelihood phylogenies were initiated by 100 maximum parsimony trees and bootstrapped with 1,000 replicates. Bayesian inference phylogenies were created using MrBayes 3.1.2 (Ronquist and Huelsenbeck 2003) using a mixed amino acid model. The analyses were ran, using default temperatures, until the two parallel chains had reached convergence (average standard deviation of split frequencies <0.01) and consisted of a minimum of 1,000,000 generations with a sampling frequency of 100. The first 25% of sampled trees were discarded as burn-in.

Phylogenies of the individual copies of each family were generated using the element termini sequences extracted through Megablast. Trees were created using maximum likelihood with RAxML and Bayesian inference with MrBayes, from nucleotide alignments created using MUSCLE. LTR retrotransposon phylogenies were generated using LTR sequences. DNA transposon and non-LTR retrotransposon phylogenies were created using 5′ inverted terminal repeat (ITR) and 3′ untranslated region (UTR) sequences, respectively. The 3′ UTR sequences were chosen for the non-LTR retrotransposon families as there were fewer 5′ UTR sequences in the genome. The maximum likelihood trees were initiated by 100 maximum parsimony trees and bootstrapped with 1,000 replicates and performed using the GTRCAT model, as recommended by the program authors. MrBayes analyses were performed using the same protocol as with the amino acid phylogenies, other than substitution model (the GTR+I+Γ nucleotide substitution model was employed).

Values of π (Nei 1987) and Tajima’s *D* (Tajima 1989) for DNA transposon and non-LTR retrotransposon families were generated from the 5′ ITR and 3′ UTR alignments used in the phylogenetics analyses. For the chromoviral families, a single LTR sequence for each insert was used to determine values of Tajima’s *D*. Individual alignments with only one LTR per insert were created for all elements, FLE, and solo LTRs to determine values of π. Population genetics statistics were calculated using DnaSP v5.10.01 (Librado and Rozas 2009).

All of the alignments, with GenBank accession numbers and Trace Read Identification Numbers, used for each analysis are present in supplementary files S3 and S4, Supplementary Material online.

## Results

### Phylogenetic Analyses of *C. owczarzaki* TE Families

**Fig. 1.—:**
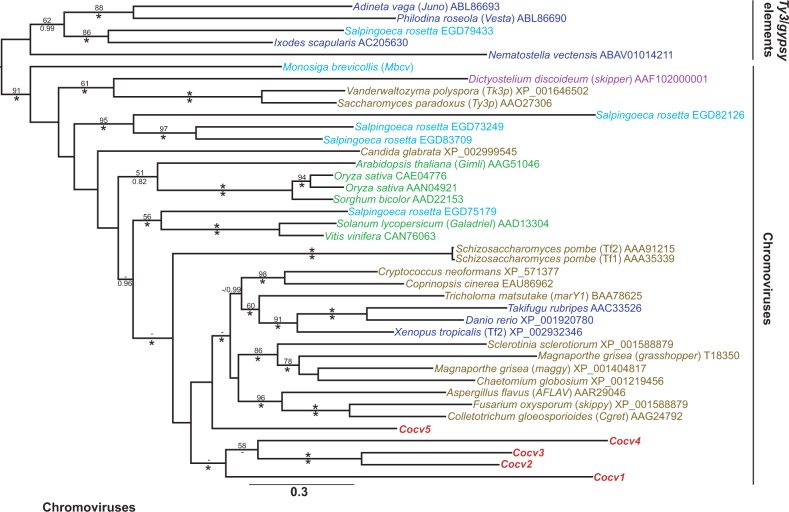
Maximum likelihood phylogeny of chromoviral Pol amino acid sequences. The phylogeny was constructed from 630 aligned amino acid positions using the PROTCAT model with the LG substitution matrix. Values for mlBPs and biPPs are shown above and below the branches, respectively. 100% mlBP and 1.00 biPP are both denoted by ‘*’. Values <50% mlBP or <0.70 biPP are denoted by “-.” *Capsaspora owczarzaki* proteins are written in red bold font, metazoan proteins are written in dark blue, choanoflagellate proteins in light blue, fungal proteins in brown, plant proteins are in dark green, and amoebozoan proteins are written in purple. The scale bar represents the number of amino acid substitutions per site.

**Fig. 2.—:**
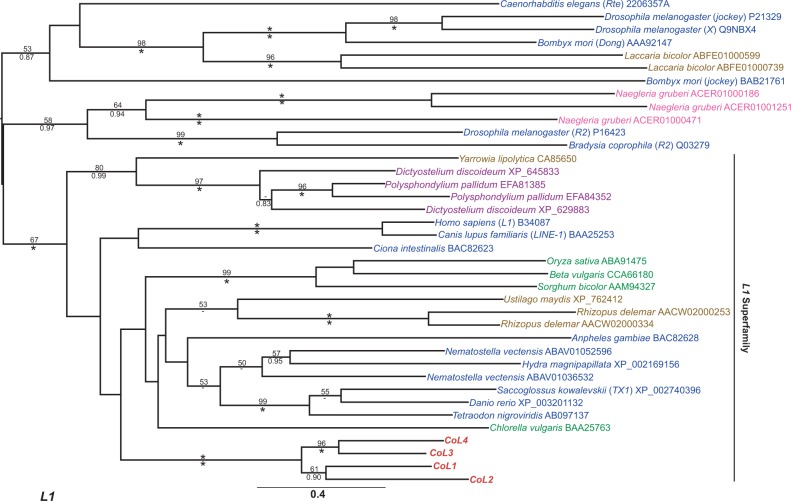
Maximum likelihood phylogeny of *L1* Pol amino acid sequences. The phylogeny was constructed from 393 aligned amino acid positions using the PROTCAT model with the LG substitution matrix. Excavate proteins are in pink font, the formatting of the tree and labels is otherwise the same as in figure 1.

**Fig. 3.—:**
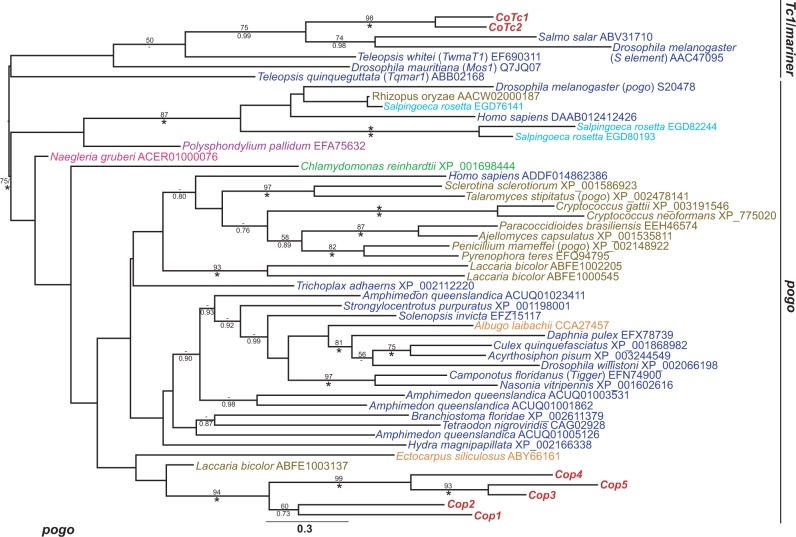
Maximum likelihood phylogeny of *pogo* Tnpase amino acid sequences. The phylogeny was constructed from 199 aligned amino acid positions using the PROTCAT model with the LG substitution matrix. Chromalveolate proteins in written in orange front, the formatting of the tree and labels is otherwise the same as in figure 2.

The *C. owczarzaki* draft genome revealed the presence of 23 families of TE in the culture ATCC 30864 (Suga et al. 2013). The analyses of Suga et al. (2013) classified the families into seven superfamilies, these being chromovirus (LTR retrotransposon), *L1* (non-LTR retrotransposon), *Cobalt*, *CACTA*, *MULE*, *pogo*, and *Tc1* (DNA transposon), but did not attempt to determine their phylogenetic relationships with TE families from other species. In order to understand the evolutionary origin of the *C. owczarzaki* TE families, phylogenies were created for each superfamily (figs. 1–3 and supplementary fig. S1*A*–D, Supplementary Material online).

Phylogenies were generated using the replication ORFs of each family, that is, the Pol of the retrotransposons and the Tnpase of the DNA transposons as they are the most highly conserved component of TE genomes. With the exception of the poorly resolved chromovirus phylogeny, all superfamily phylogenies clustered the *C. owczarzaki* families together with strong support (89–100% maximum likelihood bootstrap percentage [mlBP] and 1.00 Bayesian inference posterior probability [biPP]). In the chromoviral phylogeny, *Cocv1–4* clustered together; however, *Cocv5* has an unsupported position, clustering with Pol sequences from metazoans and fungi (fig. 1).

The grouping of the *C. owczarzaki* families together on isolated internal branches indicates that the families have a long-term evolutionary history within the filasterean lineage and have most probably radiated within it. Furthermore, all of the *C. owczarzaki* families were present in phylogenetic groups composed predominantly from other opisthokont TE families, a finding consistent with their vertical inheritance during the opisthokont radiation (figs. 1–3 and supplementary fig. S1*A–D*, Supplementary Material online).

It is becoming clear that close biological relationships, such as those of a parasite and host, may facilitate the horizontal transfer of TE families between species (Yoshiyama et al. 2001; Gilbert et al. 2010; Kuraku et al. 2012). As *C. owczarzaki* has an intimate relationship with both *B. glabrata* and *S. mansoni*, their genomes were screened for the presence of *C. owczarzaki* TEs. BLASTn screening of the *S. mansoni* genome (Berriman et al. 2009), as well as BLASTn and tBLASTn screening of the NCBI *B. glabrata* whole genome shotgun contigs and expressed sequence tags databases, failed to uncover close hits (>90% identity nucleotide/amino acid identity) of the *C. owczarzaki* TEs. These results indicate that there has been no successful horizontal transfer of TEs between *C. owczarzaki* and either species.

### Target Site Insertion Patterns of *C. owczarzaki* TEs

The integration of TEs leads to the duplication of the target sequence and, for many families, the length of the target site is highly conserved (Craig 2002). Recent work has also highlighted that many of the TE families in *D. melanogaster* possess a level of conservation in the sequences of their target sites (Nefedova et al. 2011; Linheiro and Bergman 2012). Suga et al. (2013) determined the length of target sites for 22 of the 23 TE families in the *C. owczarzaki* genome (the exception being the non-LTR retrotransposon *CoL3*) and presented here are the nucleotide compositions of the TSDs for the 22 families (supplementary fig. S2, Supplementary Material online). Within the DNA transposon families the *pogo*-like, *Tc1*-like, and *Cobalt* families all have a highly conserved, two base, TA target site. TA target sites are a common feature of many DNA transposon families (Plasterk et al. 1999); however, the *CoCACTA* and *Com* families all possess longer TSDs. The two *CACTA* families have three base TSDs, whereas the two *Com* families have nine base TSDs; however, neither superfamily shows conserved target sequences. As is commonly found with chromoviruses (Novikova et al. 2010), all five of the *C. owczarzaki* chromoviral families generate 5 base TSDs. The families do not possess conserved target sequences, but there is a general preference for guanine or cytosine (GC) in both the terminal 5′ and 3′ positions of the target sequence. Target sites were identified for total of 169 LTRs, from across all five families, and the GC content for the first and fifth positions are 66.9% and 68%, respectively. In contrast, the internal three nucleotides in the target sites are relatively GC poor (38.5%, 40.2%, and 39.6% GC content). Three non-LTR retrotransposon families had the lengths of their TSDs identified by Suga et al. (2013), these being *CoL1*, *CoL2*, and *CoL4*; however, the three families have no discernable conservation in TSD sequence (supplementary fig. S2, Supplementary Material online).

### Evidence for Recent TE Activity in the *C. owczarzaki* Genome

A number of eukaryotic species have been shown to possess families of TE that are no longer capable of transposition (Zou et al. 1995; Hellen and Brookfield 2011). The presence of multiple copies in a sequenced genome therefore does not confirm that a family is currently active within its host population. Twenty-two of the families have multiple copies in the sequenced *C. owczarzaki* genome (tables 1–3), with one family, the DNA transposon *Cobalt3*, being present as a single copy. Database mining of the *C. owczarzaki* genome and transcriptome was therefore employed to produce four lines of evidence, in the absence of observed transposition, to investigate element activity within the *C. owczarzaki* population. To this end, all terminal and flanking sequences present in the NCBI Trace Archive were extracted to identify the sequences of individual element copies. The Trace Archive was screened in favor of the assembled *C. owczarzaki* genome as TE insertions are often collapsed in draft genome assemblies.

**Table 1:** Characterization of the five identified families of LTR retrotransposon in the genome of *Capsaspora owczarzaki*

Family	Copy Number (FLE/Solo)	No. of RNASeq Reads	No. of Identical Paralogous Copies	Intraelement LTR Identity Range (%)	Total LTR Diversity (π)	FLE LTR/Solo LTR Diversity (π)	Tajima’s *D*^a^
*Cocv1*	64 (39/25)	437,027	47	99.3–100	0.075	0.011/0.160	−2.439**
*Cocv2*	33 (23/10)	22,365	21	99.3–100	0.008	0.007/0.009	−2.396**
*Cocv3*	26 (16/10)	72,753	15	100	0.021	0.012/0.033	−2.118*
*Cocv4*	17 (1/16)	80	2	100	0.174	0.000/0.184	−2.103*
*Cocv5*	22 (14/8)	62,990	4	99.7–100	0.042	0.043/0.042	−1.526

^a^Significance levels: *<0.05, **<0.01.

**Table 2:** Characterization of the four identified families of non-LTR retrotransposon in the genome of *Capsaspora owczarzaki*

Family	Observed Copy Number	No. of RNASeq Reads	No. of Identical Paralogous Copies	Noncoding Diversity 3′ UTR (π)	Tajima’s *D*^a^
*CoL1*	12	15,275	0	0.048	−0.183
*CoL2*	51	63,284	16	0.069	−1.478
*CoL3*	30	777	0	0.164	−1.925*
*CoL4*	47	49,002	5	0.102	−2.186**

^a^Significance levels: *<0.05, **<0.01.

**Table 3:** Characterization of the 14 identified families of DNA transposon in the genome of *Capsaspora owczarzaki*

Family	Observed Copy Number (5′/3′ ITR)	No. of RNASeq Reads	No. of Identical Paralogous Copies	Noncoding Diversity 5′ ITR+UTR (π)	Tajima’s *D*^a^
*Cobalt1*	17–34 (17/17)	29,065	16	0.013	0.326
*Cobalt2*	9–18 (9/9)	8,902	4	0.032	0.262
*Cobalt3*	1	805	0	—	—
*CoCACTA1*	18 (9/18)	2,629	4	0.028	0.210
*CoCACTA2*	35 (20/24)	1,344	9	0.086	n/a
*Com1*	19 (15/16)	26,930	12	0.015	1.669
*Com2*	12 (8/5)	4,941	0	0.197	−1.297
*Cop1*	28–51 (23/28)	65,112	20	0.004	−2.078*
*Cop2*	25–44 (19/25)	56,126	17	0.081	1.135
*Cop3*	15–27 (12/15)	46,819	11	0.047	−2.003*
*Cop4*	19–37 (19/18)	32,779	17	0.011	−1.405
*Cop5*	16–31 (16/15)	457	5	0.171	−1.518
*CoTc1*	42–83 (41/42)	164,264	34	0.007	−2.669***
*CoTc2*	8–14 (6/8)	1,566	0	0.022	−1.124

Note.—n/a, not applicable.

^a^Significance levels: *<0.05, ***<0.001.

#### Expression of TE Families in C. owczarzaki

**Fig. 4.—:**
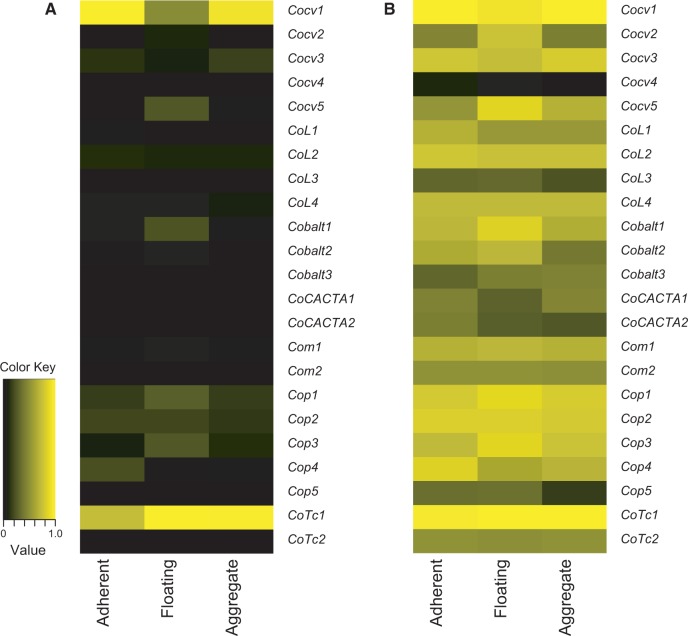
*Capsaspora owczarzaki* transposable element expression. The expression levels of transposable elements were approximated by the normalized number of RNAseq reads and shown as a heatmap. (*A*) Values were normalized by the largest expression level, which is that of *Cocv1* in adherent cells. (*B*) The color map is presented in log scale (values normalized to 0–1.0 range).

Transcription is an essential component of transposition, in order to produce catalytic proteins and, in the case of retrotransposons, RNA daughter elements. The presence of TE sequences in the RNASeq reads generated by the *C. owczarzaki* genome project (Sebé-Pedrós et al. 2013; Suga et al. 2013) would therefore provide indirect evidence that a family may be active. The complete RNASeq database contained 394,576,834 reads, of which 1,165,292 (0.3%) were of TE origin (tables 1–3 and fig. 4). Only one family, the LTR retrotransposon *Cocv4*, was essentially absent from the reads; this family presented a small number of short fragments, which did not cover the entire length of the known sequence, indicating that this family is no longer active in the sequenced culture of *C. owczarzaki*. The expression levels of the families vary by over three orders of magnitude and transcripts of *Cocv1* dominate, with 37.5% of reads being from this family. The number of RNASeq reads shows a strong positive correlation (*r* = 0.846) with the number of identical paralogous copies in the genome for each family (supplementary fig. S3, Supplementary Material online). This shows that the number of identical copies (which can be viewed as reflecting the transposition rate of a family) can be considered a good predictor of a family’s expression in *C. owczarzaki*.

RNASeq was performed on three stages in the life cycle of *C. owczarzaki*, these being an amoeboid crawling stage, a free-floating stage, and an aggregated cell stage (Sebé-Pedrós et al. 2013). Fifteen of the 23 TE families exhibited significant variation in expression level across the three stages and, of these, nine showed significantly elevated expression in a particular stage (Supporting Information File S2). Three of the four expressed LTR retrotransposon families showed significantly raised expression in one stage in the life cycle of *C. owczarzaki*; however, different families showed different expression patterns. In contrast, only one non-LTR retrotransposon family, *CoL1*, showed significant variation in expression across stages and a stage with an elevated expression level. Ten of the 14 DNA transposon families showed stage-specific expression patterns, with five (*Cobalt1*, *Com1*, *Cop1*, *Cop3*, and *Cop4*) having a preferred stage for expression.

#### Patterns of TE Nucleotide Diversity in the C. owczarzaki Genome

The second line of evidence for element activity was restricted to the LTR retrotransposons. Their LTR sequences are believed to evolve in the fashion of a molecular clock as they are identical at the time of integration and gradually diverge due to the accumulation of mutations (Bowen and McDonald 2001). Using unique TSDs, it was possible to identify the 5′ and 3′ LTRs from 40 individual inserts from across all five LTR retrotransposon families. Intraelement LTR nucleotide identity ranged between 99.3% and 100% (table 1), demonstrating that all five families have been active recently and that ancient FLE appear to be absent from the genome.

There are no analogous methods for using terminal repeats to age non-LTR retrotransposon and DNA transposon inserts. Two further methods were therefore used to obtain evidence for recent transposition in all 22 multicopy families. A number of recent studies have used Tajima’s *D* statistic to look for the signature of transposition activity (Maside et al. 2003; Sánchez-Gracia et al. 2005; Bartolomé et al. 2009; Carr et al. 2012). Under such a model, most elements in a population will have very recent common ancestry and most nucleotide variants will be present at low frequency, resulting in a negative value of *D*. If a TE family has multiple active lineages in a genome, in other words it exhibits population substructure, some nucleotide variants may be present at an intermediate frequency, which could potentially result in a positive value of *D*. It is therefore important to generate phylogenies of the individual copies of a family in order to evaluate the results of Tajima’s *D* tests. Recently, transposed elements are expected to have short terminal branches in a phylogenetic tree as daughter elements have had little time to accumulate unique mutations. The presence of identical paralogous elements can be considered as strong evidence for current element activity (tables 1–3); short terminal branches indicate that a family has recently transposed, but do not provide any direct evidence that the family is currently active in the sequenced strains. Older inserts in the genome will have accumulated numerous unique mutations and therefore be present on long terminal branches.

**Fig. 5.—:**
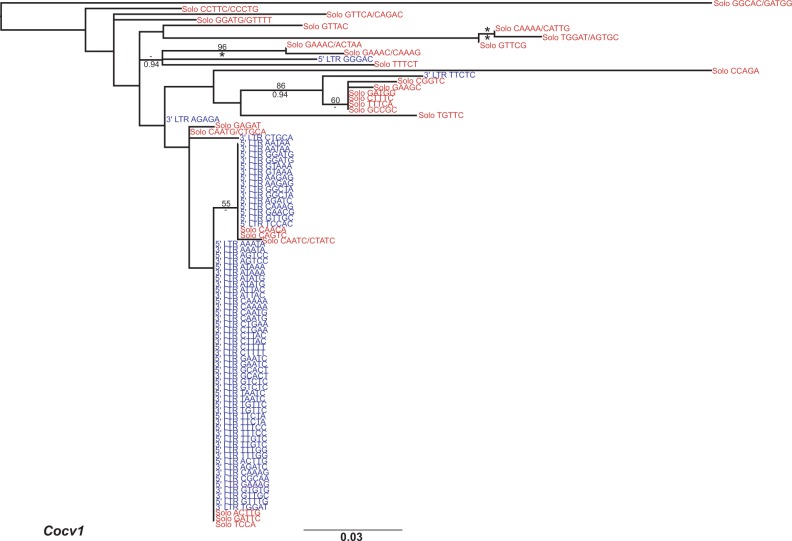
Maximum likelihood phylogenetic trees of individual element copies of *Cocv1*. The phylogeny was constructed from 171 aligned nucleotide positions using the GTRCAT model. Values for bootstrap percentages and Bayes posterior probabilities are shown above and below the branches, respectively. 100% mlBP and 1.00 biPP are both denoted by ‘*’. Values <50% mlBP or <0.70 biPP are denoted by “-.” 5′ and 3′ LTR sequences are shown in blue, solo LTR sequences are written in red. Terminal nodes are labeled with the flanking DNA sequence of the insert. The scale bar represents the number of nucleotide substitutions per site.

All of the retrotransposon families exhibited negative values for *D*, consistent with all such families having recently undergone transposition; however, the deviation from neutral expectation was not significant for the LTR retrotransposon *Cocv5*, as well as the non-LTR retrotransposons *CoL1* and *CoL2* (tables 1 and 2). In phylogenies generated from LTR sequences the five chromoviral families all harbor identical paralogous copies, confirming that they have all undergone very recent transposition in the *C. owczarzaki* genome (fig. 5 and supplementary fig. S4*A–D*, Supplementary Material online). Within the non-LTR retrotransposons all four families show copies with high nucleotide identity (>98.5% nucleotide identity), again highlighting that the families have recently been active; however, only *CoL2* and *CoL4* show identical nonallelic copies (supplementary fig. S4*E–H*, Supplementary Material online).

Values for Tajima’s *D* could not be determined for *Cobalt3* and *CoCACTA2*; the former only has a single copy in the genome, whereas the latter has too many overlapping indels to allow *D* to be calculated. However seven of the remaining 12 DNA transposon families produced negative *D* values. Significantly negative values of *D* were obtained for *Cop1*, *Cop3*, and *CoTc2*, whereas positive values of *D* were produced for *Cobalt1*, *Cobalt2*, *CoCACTA1*, *Com1*, and *Cop2* (table 3). The phylogenies of these latter five families show multiple identical sequences, consistent with their current transposition (supplementary fig. S4*I–K*, *M*, and *P*, Supplementary Material online). The families also have deep internal branching, a result of their population subdivision, which accounts for the positive values of *D*. Only three of the DNA transposon families do not show multiple copies with identical sequences, these being the single copy *Cobalt3* as well as *Com2* and *CoTc2* (supplementary fig. S4*N* and *U*, Supplementary Material online). However all multicopy DNA transposon families have multiple inserts with high nucleotide (≥99%) identity, highlighting their recent transposition.

**Fig. 6.—:**
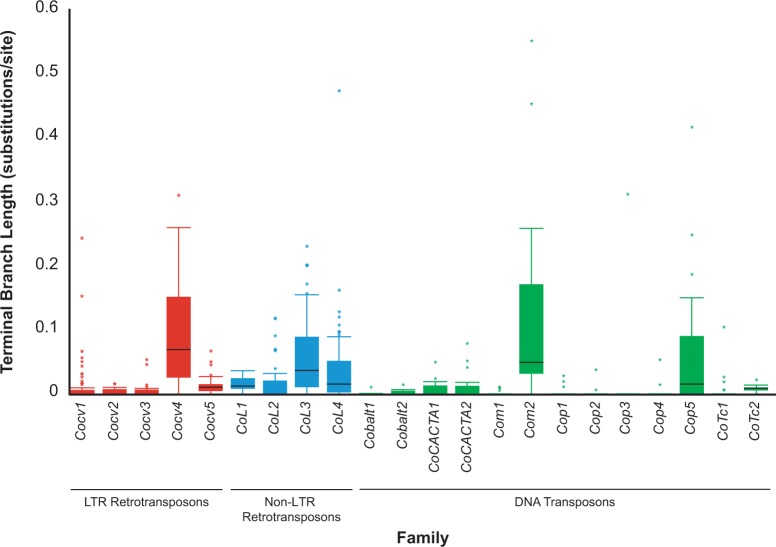
Terminal branch lengths of the 22 multicopy transposable element families in the *Capsaspora owczarzaki* genome. LTR retrotransposon, non-LTR retrotransposon, and DNA transposon families are represented by red, blue, and green boxes, respectively. Branch lengths for full length LTR retrotransposons were taken from the 5′ LTR when this was present in the phylogeny; in its absence, the 3′ LTR was used. The filled boxes denote the interquartile range and the horizontal dark line represents the median branch length. The whiskers highlight 1.5 times the interquartile range from the median and the asterisks represent branch lengths outside this range.

Bergman and Bensasson (2007) used terminal branch length as a proxy for retrotransposon age in the genome of *D. melanogaster*, showing that LTR families were composed from younger individuals than non-LTR families. There is no such dichotomy in the *C. owczarzaki* genome, rather the phylogenies of all TE classes show that the *C. owczarzaki* genome is dominated by young copies. However 13 of the families contain older inserts (defined as copies with a branch length ≥0.05 substitutions/site—fig. 6) indicating that some individual elements can persist for a long time in the *C. owczarzaki* genome.

### *Capsaspora owczarzaki* TE Families Show Differing Population Histories

TE families can be considered to have their own life cycle within their host’s genome (Brookfield 2005). The family has an origin (either through horizontal transfer between hosts or TE “speciation” within a single host population) and subsequently proliferates in the genome. Individual elements in the new family accumulate null mutations, until no more functional copies exist and the family finally becomes a genomic fossil incapable of further transposition. The phylogenies of the 22 multicopy TE families show markedly different topologies (fig. 5 and supplementary fig. S4*A–U*, Supplementary Material online) and levels of nucleotide diversity within the families, based upon π, vary by over an order of magnitude (tables 1–3). It is clear therefore that the families exhibit different population dynamics within the *C. owczarzaki* genome.

Within the chromoviral families *Cocv1* (fig. 5) and *Cocv3* (supplementary fig. S4*B*, Supplementary Material online) both contain groups of long branch, presumably ancient, solo LTRs as well as clades of shorter branched sequences made up from both solo LTRs and FLE. In contrast there are no long branch copies of *Cocv2* (supplementary fig. S4*A*, Supplementary Material online) and the phylogeny has a star-like appearance, highlighting a recent common origin of all copies in the genome.

Carr et al. (2008) reported a greater level of nucleotide diversity among paralogous solo LTRs compared with LTRs from FLE in the choanoflagellate *M. brevicollis* as solo LTRs can persist in a population for a longer period of time. A similar situation exists in the case of *Cocv1*, *Cocv3*, and *Cocv4* (table 1). In *Cocv2* and *Cocv5*, there are similar levels of nucleotide diversity in both solo LTRs and LTRs from FLE; however, their phylogenies highlight that the families have different population histories. *Cocv2* presents a star phylogeny with very little sequence divergence between copies (supplementary fig. S4*A*, Supplementary Material online). In contrast, *Cocv5* shows a higher degree of population substructure than other LTR retrotransposon families, with multiple apparently active lineages in the genome (supplementary fig. S4*D*, Supplementary Material online). Furthermore, no two of the 14 full length *Cocv5* elements in the genome have identical sequences (supplementary fig. S4*D*, Supplementary Material online). This suggests that the family may have a lower recent rate of transposition in comparison to *Cocv1–3*, which all possess large numbers (>15) of identical full length copies. A putative low transposition rate is however not reflected by the expression level of *Cocv5*, which is the sixth most abundant TE family in the RNASeq database (tables 1–3). Matsuda and Garfinkel (2009) showed that antisense RNA copies of *Ty1* in *Saccharomyces* can inhibit transposition; therefore, the high expression of *Cocv5* and its putative low transposition rate are not incompatible.

In contrast to the other chromoviral families, which have a greater number of FLE than solo LTRs, *Cocv4* consists mainly of long branched, presumably old, solo LTRs (supplementary fig. S4*C*, Supplementary Material online) and harbors the greatest level of nucleotide diversity within the chromoviruses (table 1). A single FLE is present in the genome. This element appears to be the product of a recent transposition event as no nucleotide substitutions have occurred in either of the LTRs. The element, and therefore the entire family, however appears to be no longer capable of transposition in the sequenced strains of *C. owczarzaki* as the 3′ LTR is truncated to 58 bp in length by a large deletion. The lack of element activity is consistent with the dearth of RNASeq reads for this family, which only cover a small proportion of the *Cocv4* genome (data not shown).

Within the non-LTR retrotransposons, *CoL2* and *CoL4* show similarities in their phylogenies in that both families contain long branched inserts as well as clusters of short branch, presumably younger, copies (supplementary fig. S4*F* and *H*, Supplementary Material online). The two families also show multiple identical copies. The data indicate that both are old families that are currently transposing within the *C. owczarzaki* population. In contrast, although they both possess highly similar inserts, there are no identical nonallelic copies of either *CoL1* or *CoL3* (supplementary fig. S4*E* and *G*, Supplementary Material online). The data therefore suggest that both families have been active in the recent evolutionary history of *C. owczarzaki*; however, their transposition may have currently ceased. Furthermore, both *CoL1* and *CoL3* have considerably lower expression levels than those of *CoL2* and *CoL4* (table 2).

Within the DNA transposon families the level of nucleotide diversity varies by over an order of magnitude (table 3). This is due to some families showing either deep or complex subdivision (e.g., *Com2*, *Cop2*, *Cop5*—supplementary fig. S4*N*, *P*, and *S*, Supplementary Material online), whereas other families (*Cop1* and *CoTc1*—supplementary fig. S4*O* and *T*, Supplementary Material online) show limited subdivision with most copies having a single recent common ancestry. Seven of the DNA transposon families (*Cobalt1–2*, *CoCACTA1*, *Com1*, *Cop1–2*, and *CoTc2*—supplementary fig. S4*I*, *J*, *M*, *O*, *P*, and *U*, Supplementary Material online) are composed entirely from young elements (i.e., copies with a branch length <0.05 substitutions/site). However, all of these families show internal branching and population subdivision; highlighting that the families are older than the current population of copies in the genome.

## Discussion

### TE Family Diversity in the *C. owczarzaki* Genome

*Capsaspora owczarzaki* is the second holozoan protist, after *M. brevicollis*, to have a survey undertaken of its TEs. The TE complement of *C. owczarzaki* shows some similarities, but also many differences, to that of *M. brevicollis* and therefore affords us a greater understanding of TE evolution within the holozoans.

The most obvious difference between the two species is the greater diversity of families present in the genome of *C. owczarzaki*, in terms of both the number of families and the different classes of element present. *Monosiga brevicollis* possesses a very limited range of TEs, with only three families, all of which are LTR retrotransposons (Carr et al. 2008). This contrasts sharply with the 23 families present in the *C. owczarzaki* genome, which can be assigned to seven superfamilies of DNA transposon, LTR retrotransposon, and non-LTR retrotransposon. The genome sequence of *C. owczarzaki* was produced from a polymorphic and outbred culture, which suggests that we have captured much of the TE family diversity in this species, but it remains possible that additional families are present in the full population. The low number of families present in *M. brevicollis* would appear to be a result of major element loss and, as suggested by Carr et al. (2008), may be atypical for choanoflagellates. The closely related choanoflagellate *S. rosetta* possesses TEs from four of the superfamilies present in the *C. owczarzaki* genome (chromovirus, *Cobalt*, *pogo,* MULE; see figs. 1 and 3, supplementary fig. S1*A* and *D*, Supplementary Material online) and a third choanoflagellate, *Monosiga* sp. (ATCC 50635), has also been shown to possess DNA transposon, LTR and non-LTR retrotransposon families (Carr et al. 2008).

All seven superfamilies present in the *C. owczarzaki* genome are nested within clades composed predominantly of opisthokont families, consistent with their inheritance within the group by vertical transmission. This strongly suggests that the ancestral opisthokont also possessed a diverse complement of TEs and that widespread element loss and lineage sorting have occurred during the opisthokont radiation. The presence of all of the seven superfamilies in the genomes of metazoans indicates that the superfamilies were also probably present in the last common ancestor of Metazoa. These data are of particular importance in beginning to determine the evolution of TEs in Holozoa. None of the families present in the *M. brevicollis* genome cluster with holozoan sequences (Carr et al. 2008), so they were not informative for studies in the ancestral TE composition of either Holozoa or Metazoa. However, reconstructing the deep evolutionary history of TE superfamilies can be a difficult process, due to their rapid rate of evolution (Peterson-Burch and Voytas 2002) which often results in unresolved phylogenetic trees. As a result, phylogenies are often consistent with both vertical and horizontal inheritance. The LTR retrotransposon phylogeny (fig. 1) shows that all three screened species of holozoan protist (*C. owczarzaki*, *M. brevicollis*, and *S. rosetta*) possess chromovirus families. Chromoviruses are also a common feature of fungal genomes and it has been suggested that they were a component of the ancestral opisthokont genome (Kordiš 2005; Carr et al. 2008). This makes their apparent paucity in metazoan genomes, where they appear to be restricted to a small number of vertebrate lineages (Kordiš 2005), something of an enigma. The chromoviral phylogeny has a poorly resolved backbone, so cannot rule out the vertical transmission and subsequent loss of the superfamily in the majority of metazoan lineages. Nevertheless, the phylogeny does not cluster the chromoviruses from Metazoa with those of their sister group, the choanoflagellates, as would be expected under vertical inheritance (fig. 1). Although the relationship is poorly supported, the metazoan chromoviruses are nested within those from the Dikarya fungi; therefore, the phylogeny is also consistent with the potential horizontal transfer of chromoviruses to an ancestral vertebrate from a fungal lineage. The phylogeny is also consistent with the opisthokont last common ancestor having a highly diverse suite of chromovirus families, which have subsequently segregated in different lineages due to stochastic loss.

The increasing volume of available genomic data from opisthokont protist lineages, for example, from multiple ichthyosporean, filasterean and nuclearioid taxa (Ruiz-Trillo, Burger et al. 2008), is likely to soon bridge the large evolutionary distances between the TE families placed in the current phylogenies. These increased data should provide greater resolution to phylogenetic analyses and may allow questions on the inheritance of superfamilies within the opisthokonts to be answered.

### TE Activity in the *C. owczarzaki* Genome

All three families in the *M. brevicollis* genome appear to be currently active (Carr et al. 2008). In contrast, *C. owczarzaki* has one putatively nonfunctional family in *Cocv4* and a further five families (*Cobalt3*, *CoL1*, *CoL3*, *Com2,* and *CoTc2*) that show no phylogenetic evidence for current transposition. Inactive TE families are a common feature in a broad range of opisthokont genomes (Belshaw et al. 2005; Bergman and Bensasson 2007; Carr et al. 2012); therefore, the presence of such families in the *C. owczarzaki* genome is not surprising. Furthermore, the TE families in the *C. owczarzaki* genome appear to be at different stages in their life cycles. The six families listed earlier appear to be either nonfunctional or potentially in the process of losing activity, whereas the majority of families appear to be active, long-term inhabitants of the genome. *Cocv2* however appears to be a very young family. The family has no old copies present in the genome, has a very low level of nucleotide diversity, and presents a star like phylogeny (supplementary fig. S4*A*, Supplementary Material online and table 1). In these respects it is very similar to *Ty2*, which has recently undergone a horizontal transfer from *S. mikatae* into the genome of *S. cerevisiae* (Carr et al. 2012). Similarly, the fact that there is only a single copy of *Cobalt3* in the genome is consistent with its own recent arrival in the genome. The single *Cobalt3* element is expressed in *C. owczarzaki* cells, albeit it at a low level, and encodes a conserved Tnpase protein (Suga et al. 2013) suggesting that the family has the potential to invade the genome. *Cocv2* and *Cobalt3* may therefore be new arrivals into the *C. owczarzaki* genome; however this cannot be confirmed in the absence of sequences from putative donor species and, despite a lack of evidence to show this, both families could be long-term inhabitants in the genome.

The TEs present in *M. brevicollis* and *C. owczarzaki* genome show a similarly low copy number, with all families harboring <100 copies (tables 1–3). This is in sharp contrast to many metazoan taxa, where families may be present in many thousands of copies (Robertson and Lampe 1995; Smit and Riggs 1996). The low copy numbers observed are likely to be, in part, due to the large population sizes of protists (Finlay and Fenchel 2004), which will facilitate more efficient purifying selection against TE induced deleterious mutations. It may also reflect a requirement of single celled organisms to maintain a streamlined genome in order to efficiently undergo rapid nuclear replication, cell division, and reproduction.

Further similarities between *C. owczarzaki* and *M. brevicollis* are seen with regards to their complement of LTR retrotransposons. In both species, irrespective of the overall age of the families, FLE are always young, with no single FLE showing more than 2.5% divergence between its two LTRs. This trait however is seen in a broad range of eukaryotes (Bowen and McDonald 2001; Gorinšek et al. 2004; Kordiš 2005; Xu et al. 2006), suggesting that full length LTR retrotransposons tend to be highly deleterious and rapidly removed from populations, either by the action of purifying selection or LTR–LTR recombination.

The apparent high rate of element turnover in the *C. owczarzaki* genome is not restricted to LTR retrotransposon families. The multicopy families are predominantly composed from short branched, presumably young copies, highlighting that the majority of copies has recently integrated into the genome. The presence of a small number of ancient inserts (fig. 6) and deep internal branching within phylogenies show that most families are long-term inhabitants of the genome. Therefore, it would appear that the majority of TE insertions is deleterious in the *C. owczarzaki* genome and that natural selection operates efficiently to remove individual copies. However, recent work by Blumenstiel et al. (2014) showed that the young age of many TEs copies in *D. melanogaster* is consistent with recent bursts of transposition, rather than continual negative selection and element turnover. There therefore is a possibility that the skew toward young elements in *C. owczarzaki* is a result of bursts of transposition in the families present in the genome. Testing this alternative scenario will require the discovery of additional populations of *C. owczarzaki* in order to determine the allele frequency spectrum of insertions. A current obstacle to population studies in *C. owczarzaki* is the lack of multiple cultures as only one laboratory culture has been established since the discovery of the organism in 1977. Until additional populations are isolated, it will be difficult to distinguish constant element turnover and recent bursts of transposition for the families present in *C. owczarzaki*.

Recent work on *S. cerevisiae* (Carr et al. 2012) has shown that long branch, ancient copies are predominantly fixed in the population, whereas short branch, young copies of TEs tend to be polymorphic. If this observation holds for *C. owczarzaki*, the TE allele frequency spectrum can be predicted to be highly variable within the host population as most copies appear to be young. Under this scenario, TEs can be expected to be a major source of genetic diversity in the *C. owczarzaki* population.

The use of RNASeq reads presented here gives an additional insight into the biology of protistan TEs. There is a strong positive correlation between expression level and the number of newly transposed elements. Although such a relationship may be expected, posttranslational regulation (Matsuda and Garfinkel 2009) may uncouple the rates of element expression and transposition. Indeed the relationship between expression and transposition does not hold for all families as the LTR retrotransposon *Cocv5* and the non-LTR retrotransposon *CoL4* have high expression levels yet possess relatively few recently integrated elements.

The stage-specific transcriptome data presented here indicate that there are sophisticated regulatory interactions between *C. owczarzaki* and its TE complement. The majority of families shows a level of variation in expression between different stages, with nine families showing significantly elevated expression in one stage in the life cycle. Six of the families show significantly elevated expression in the floating stage, a stage seen in the laboratory as a response to overcrowding in cultures. This elevated expression may be a result of the host cell being under stress, due to overcrowding, as numerous TE families have been shown to be activated by host stress (Capy et al. 2000). It seems unlikely that a stress response is the sole cause of elevated TE expression as other families are more highly expressed in the amoeboid adherent and aggregate stages.

The survey completed here sheds further light on the evolution of TEs within holozoan protist genomes. Data from additional genomes are required before general trends, if indeed there are any across such a broad range of organisms, can be identified. The relative simplicity of sequencing small protist genomes compared with those of metazoans should, in the near future, provide insights into the inheritance and population dynamics of protistan TEs at both the species and genome level.

## Supplementary Material

Supplementary files S1–S4 and figures S1–S4 are available at *Genome Biology and Evolution* online (http://www.gbe.oxfordjournals.org/).

Supplementary Data
