# Supplementary material for: The Holozoan Capsaspora owczarzaki Possesses a Diverse Complement of Active Transposable Element Families
Source: Genome Biol Evol. 2014 Apr 2;6(4):949–63. doi: 10.1093/gbe/evu068 (PMC4007536; doi:10.1093/gbe/evu068)
Supplement: Supplementary Data [file supp_6_4_949__index.html]

The Holozoan Capsaspora owczarzaki Possesses a Diverse Complement of Active Transposable Element Families — Supplementary Data 

# The Holozoan *Capsaspora owczarzaki* Possesses a Diverse Complement of Active Transposable Element Families

## Supplementary Data

files

**Files in this Data Supplement:**

- Supplementary Data - zip file
