# Supplementary material for: The Holozoan Capsaspora owczarzaki Possesses a Diverse Complement of Active Transposable Element Families
Source: Genome Biol Evol. 2014 Apr 2;6(4):949–63. doi: 10.1093/gbe/evu068 (PMC4007536; doi:10.1093/gbe/evu068)
Supplement: Supplementary Data [file supp_evu068_suppl_data.zip › Figure S2.pdf]

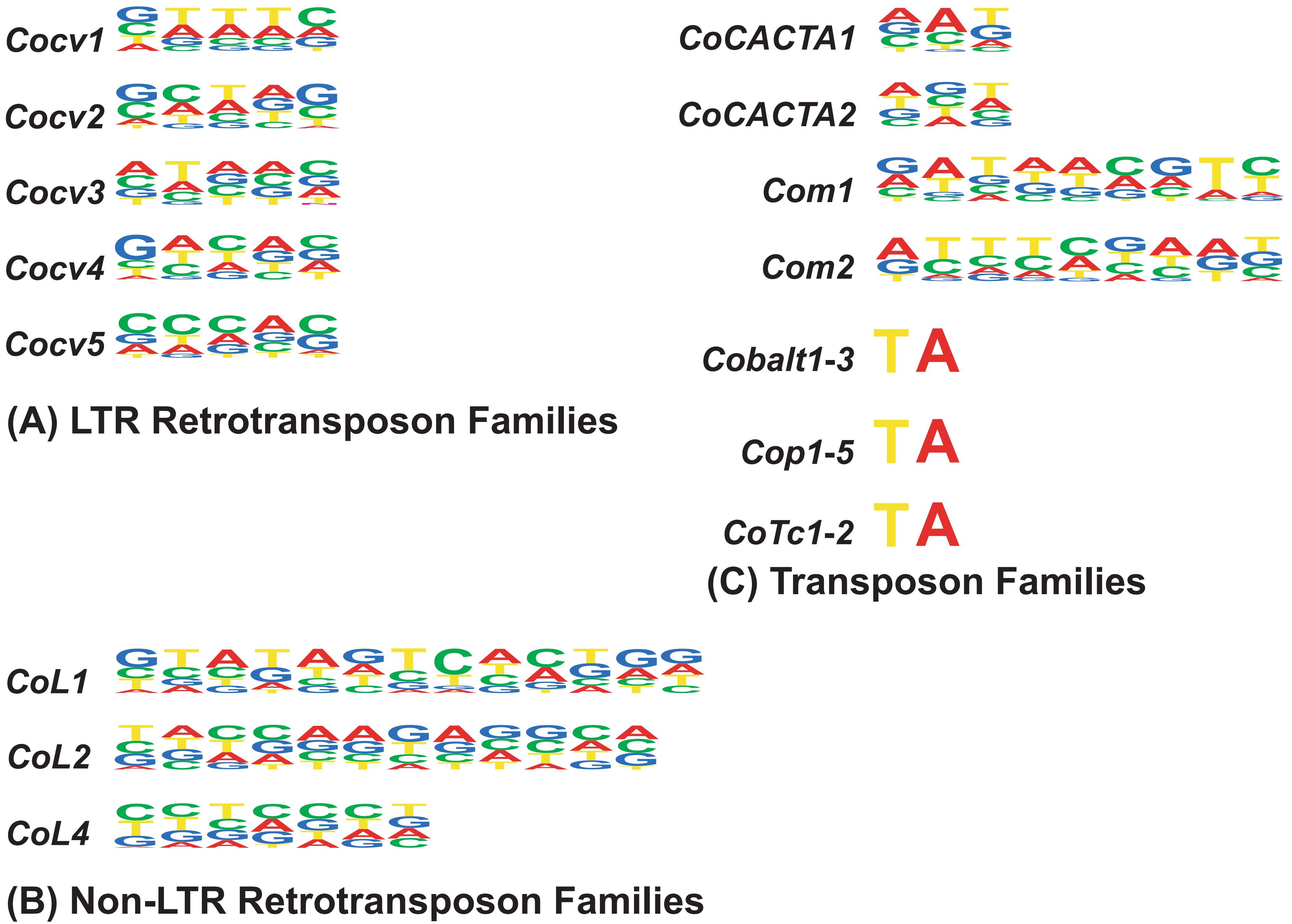
Cocv1
Cocv2
Cocv3
Cocv4
Cocv5
(A) LTR Retrotransposon Families
CoCACTA1
CoCACTA2
Com1
Com2
Cobalt1-3
Cop1-5
CoTc1-2
(C) Transposon Families
CoL1
CoL2
CoL4
(B) Non-LTR Retrotransposon Families
