# Supplementary material for: The Holozoan Capsaspora owczarzaki Possesses a Diverse Complement of Active Transposable Element Families
Source: Genome Biol Evol. 2014 Apr 2;6(4):949–63. doi: 10.1093/gbe/evu068 (PMC4007536; doi:10.1093/gbe/evu068)
Supplement: Supplementary Data [file supp_evu068_suppl_data.zip › Figure S4.pdf]

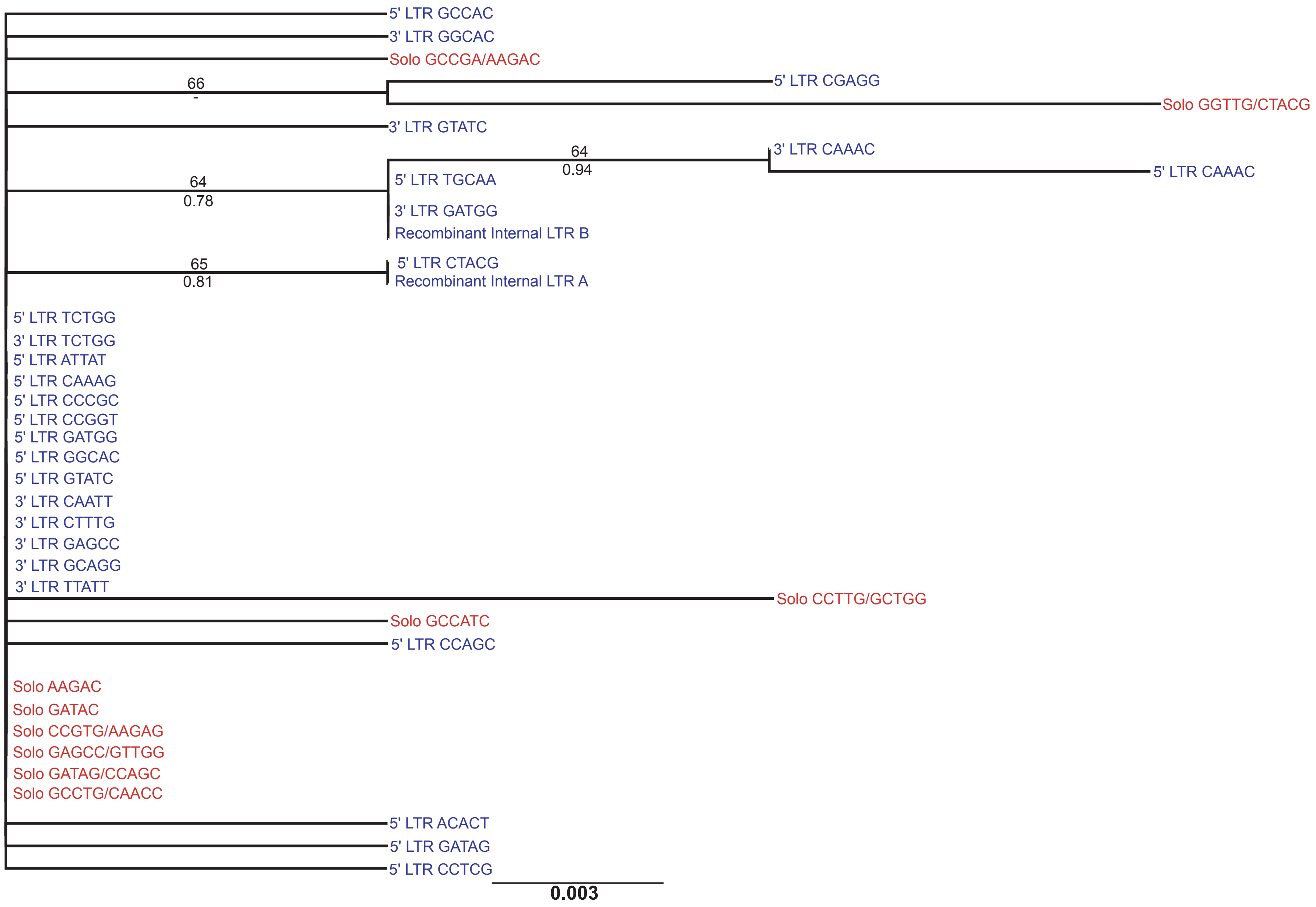
5' LTR GCCAC
3' LTR GGCAC
Solo GCCGA/AAGAC
66
-
5' LTR CGAGG
Solo GGTTG/CTACG
3' LTR GTATC
64
0.94
3' LTR CAAAC
5' LTR CAAAC
64
0.78
5' LTR TGCAA
3' LTR GATGG
Recombinant Internal LTR B
65
0.81
5' LTR CTACG
Recombinant Internal LTR A
5' LTR TCTGG
3' LTR TCTGG
5' LTR ATTAT
5' LTR CAAAG
5' LTR CCCGC
5' LTR CCGGT
5' LTR GATGG
5' LTR GGCAC
5' LTR GTATC
3' LTR CAATT
3' LTR CTTTG
3' LTR GAGCC
3' LTR GCAGG
3' LTR TTATT
Solo CCTTG/GCTGG
Solo GCCATC
5' LTR CCAGC
Solo AAGAC
Solo GATAC
Solo CCGTG/AAGAG
Solo GAGCC/GTTGG
Solo GATAG/CCAGC
Solo GCCTG/CAACC
5' LTR ACACT
5' LTR GATAG
5' LTR CCTCG
0.003

(A) ***Cocv2***

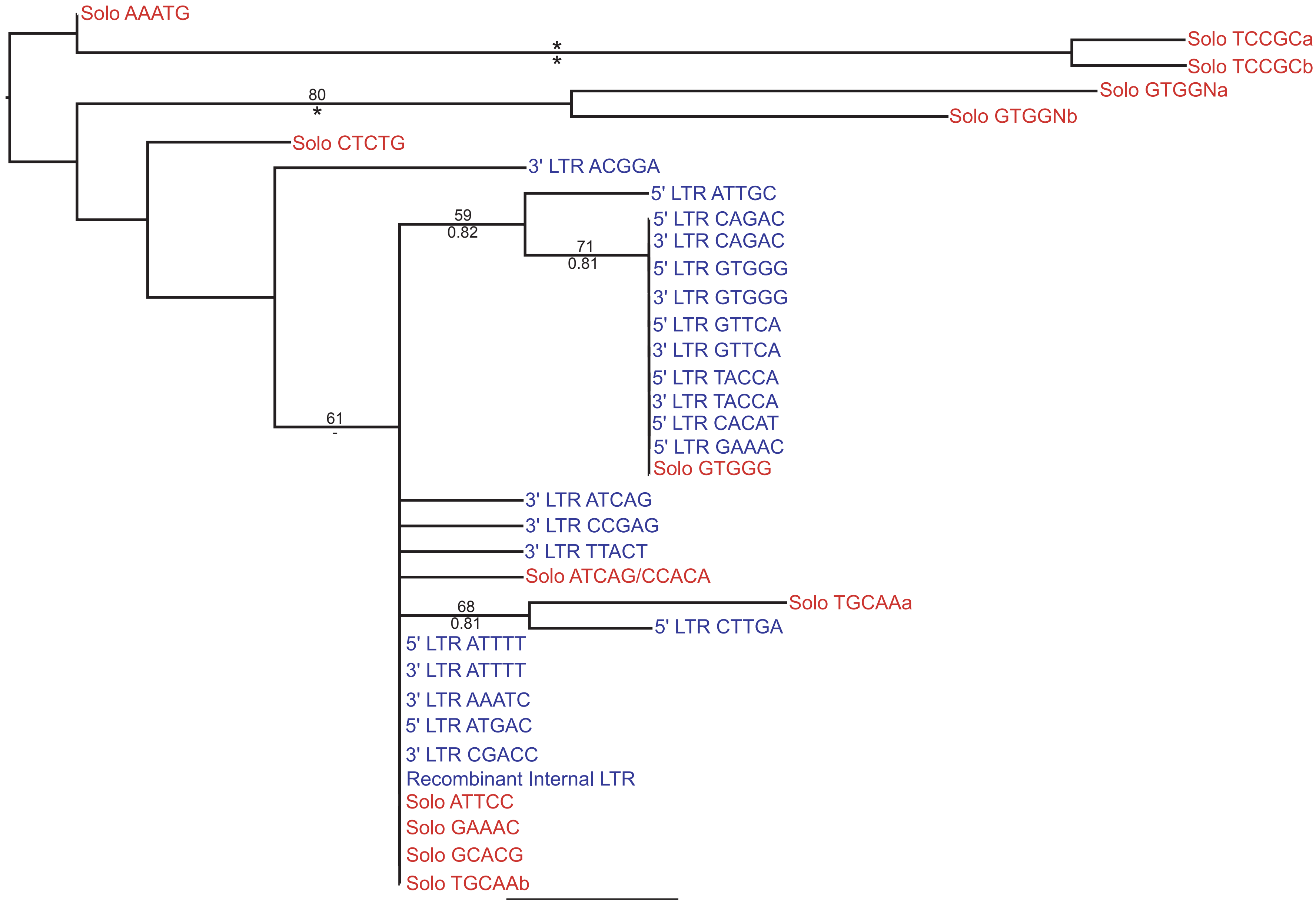

Solo AAATG
Solo TCCGCa
Solo TCCGCb
*
*
80
*
Solo GTGGNa
Solo GTGGNb
Solo CTCTG
3' LTR ACGGA
59
0.82
5' LTR ATTGC
71
0.81
5' LTR CAGAC
3' LTR CAGAC
5' LTR GTGGG
3' LTR GTGGG
5' LTR GTTCA
3' LTR GTTCA
5' LTR TACCA
3' LTR TACCA
5' LTR CACAT
5' LTR GAAAC
Solo GTGGG
61
-
3' LTR ATCAG
3' LTR CCGAG
3' LTR TTACT
Solo ATCAG/CCACA
68
0.81
Solo TGCAAa
5' LTR CTTGA
5' LTR ATTTT
3' LTR ATTTT
3' LTR AAATC
5' LTR ATGAC
3' LTR CGACC
Recombinant Internal LTR
Solo ATTCC
Solo GAAAC
Solo GCACG
Solo TGCAAb
0.008

(B) ***Cocv3***

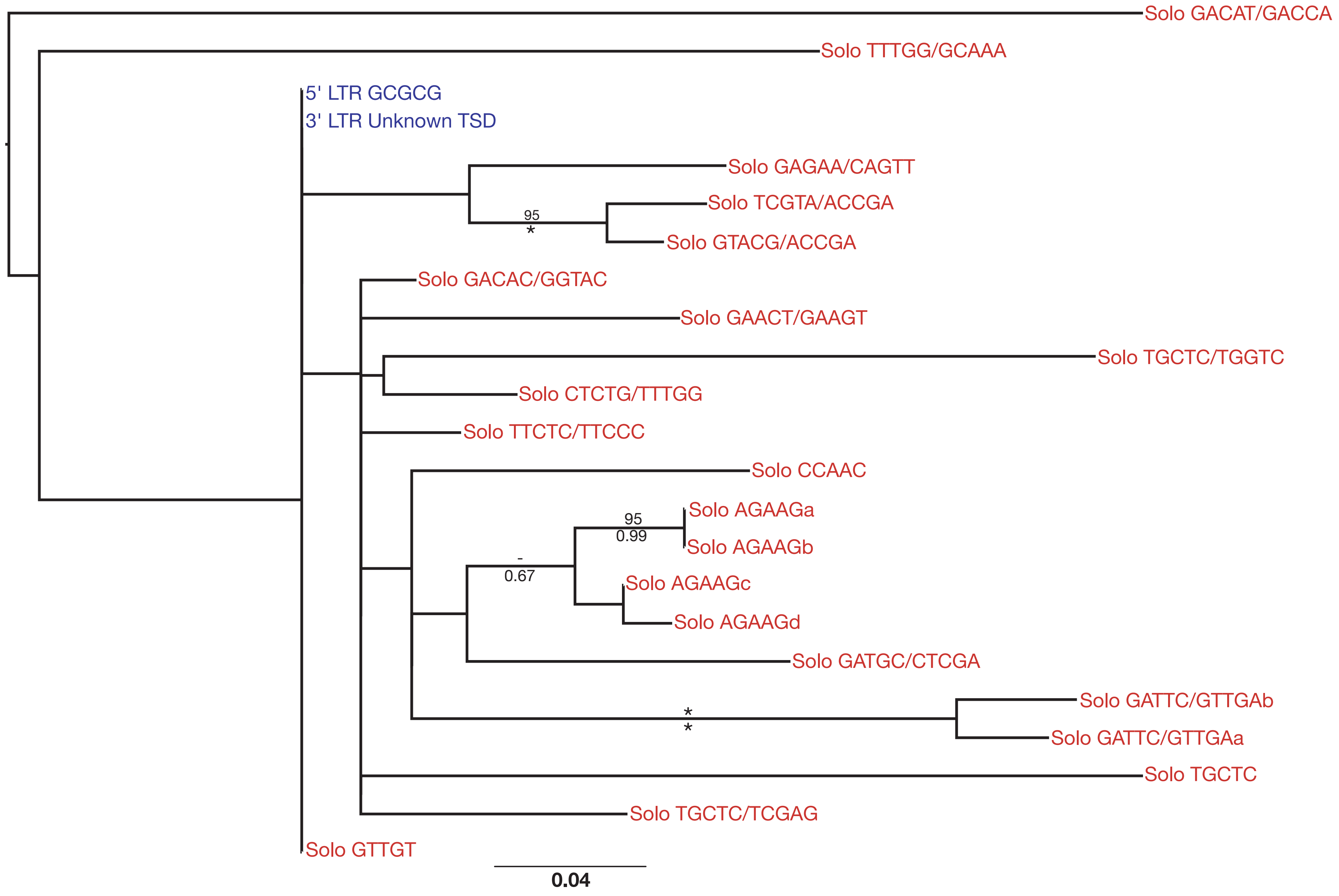
Solo GACAT/GACCA
Solo TTTGG/GCAAA
5' LTR GCGCG
3' LTR Unknown TSD
Solo GAGAA/CAGTT
Solo TCGTA/ACCGA
95
*
Solo GTACG/ACCGA
Solo GACAC/GGTAC
Solo GAACT/GAAGT
Solo TGCTC/TGGTC
Solo CTCTG/TTTGG
Solo TTCTC/TTCCC
Solo CCAAC
Solo AGAAGa
95
0.99
Solo AGAAGb
-
0.67
Solo AGAAGc
Solo AGAAGd
Solo GATGC/CTCGA
Solo GATTC/GTTGAb
*
*
Solo GATTC/GTTGAa
Solo TGCTC
Solo TGCTC/TCGAG
Solo GTTGT
0.04

(C) ***Cocv4***

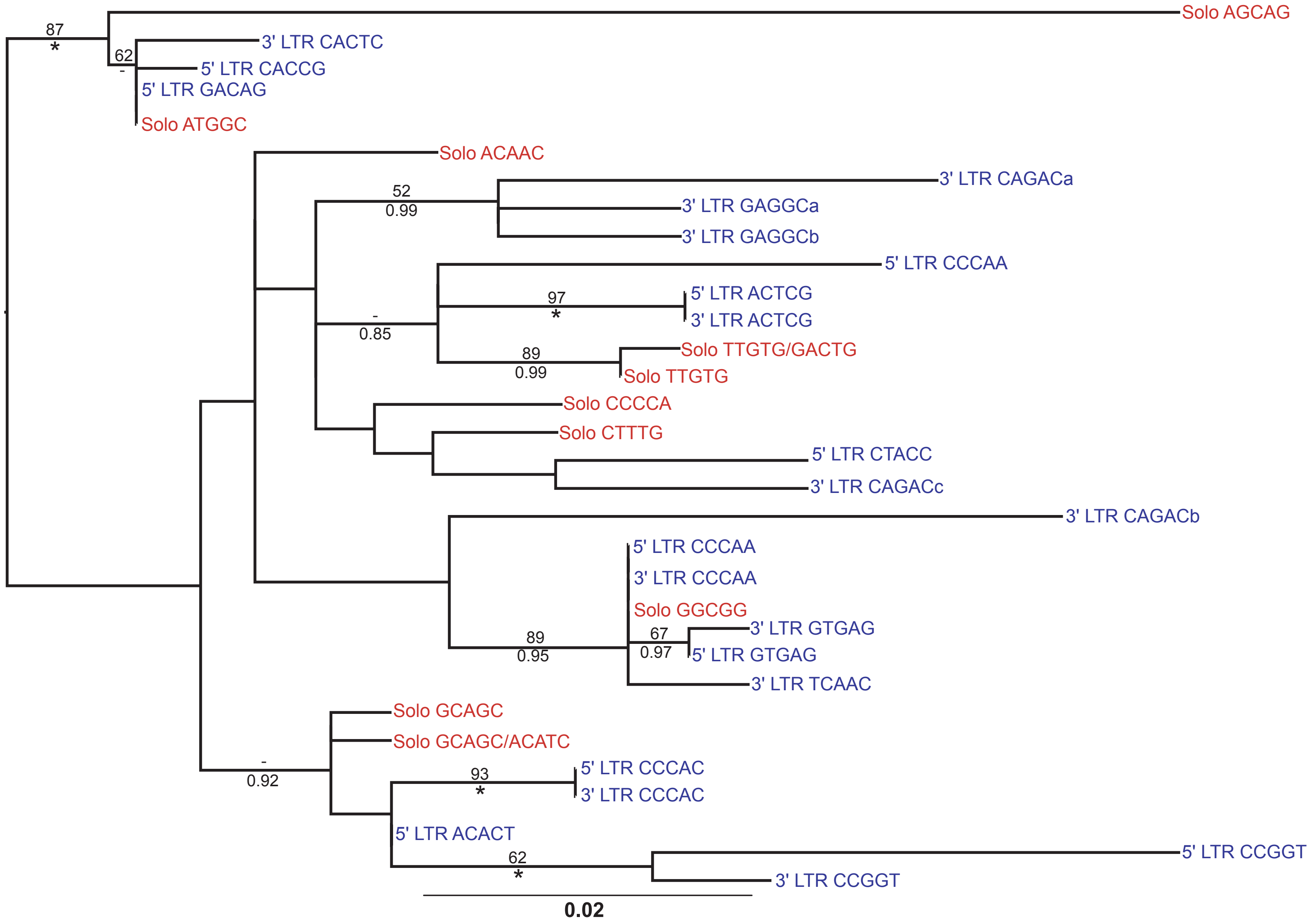

Solo AGCAG
87
*
3' LTR CACTC
62
-
5' LTR CACCG
5' LTR GACAG
Solo ATGGC
Solo ACAAC
3' LTR CAGACa
52
0.99
3' LTR GAGGCa
3' LTR GAGGCb
5' LTR CCCAA
97
*
5' LTR ACTCG
3' LTR ACTCG
-
0.85
89
0.99
Solo TTGTG/GACTG
Solo TTGTG
Solo CCCCA
Solo CTTTG
5' LTR CTACC
3' LTR CAGACc
3' LTR CAGACb
5' LTR CCCAA
3' LTR CCCAA
Solo GGCGG
89
0.95
67
0.97
3' LTR GTGAG
5' LTR GTGAG
3' LTR TCAAC
Solo GCAGC
Solo GCAGC/ACATC
-
0.92
93
*
5' LTR CCCAC
3' LTR CCCAC
5' LTR ACACT
62
*
5' LTR CCGGT
3' LTR CCGGT
0.02

(D) *Cocv5*

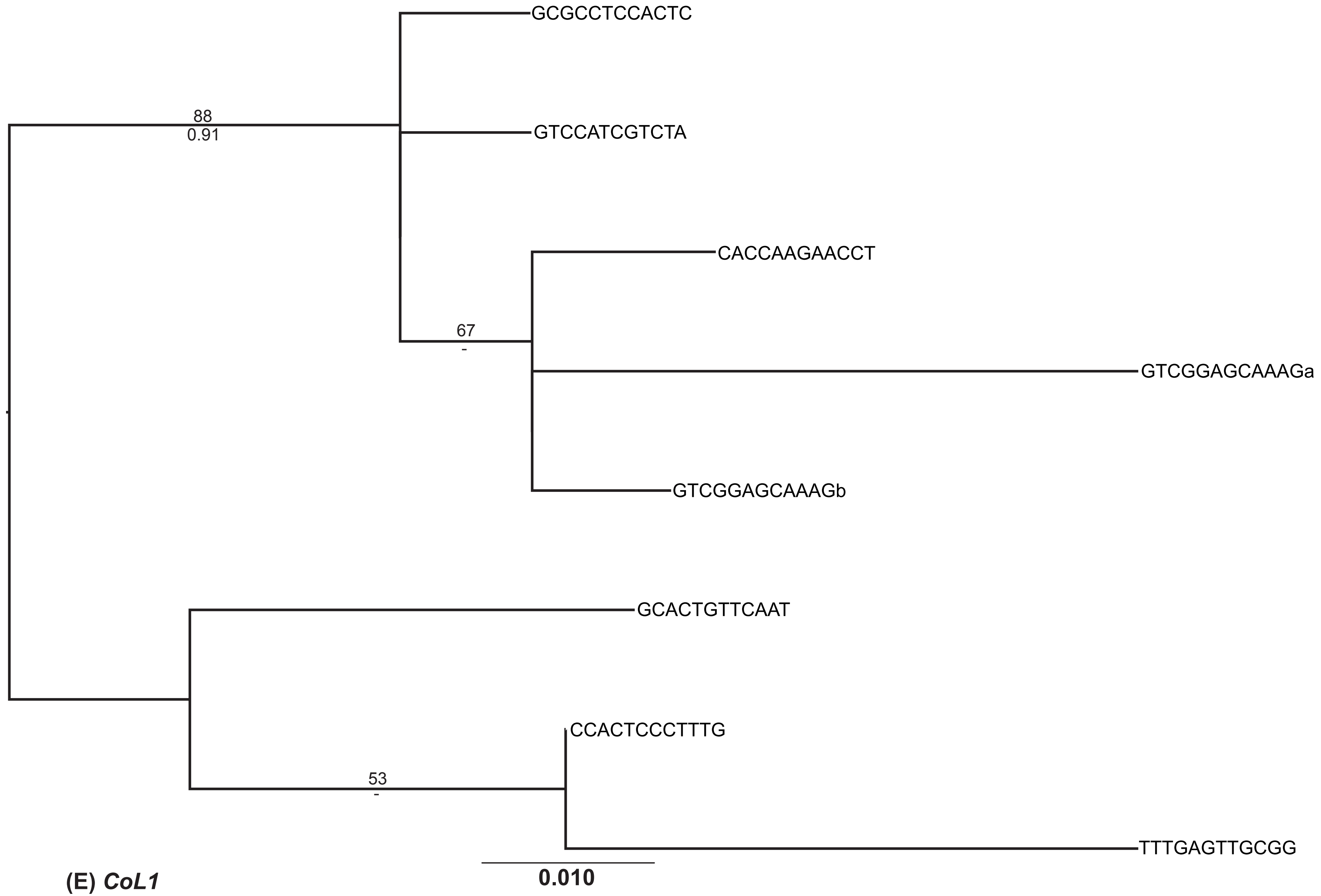

GCGCCTCCACTC
88
0.91
GTCCATCGTCTA
CACCAAGAACCT
67
-
GTCGGAGCAAAGa
GTCGGAGCAAAGb
GCACTGTTCAAT
CCACTCCCTTTG
53
-
TTTGAGTTGCGG
0.010

(E) ***CoL1***

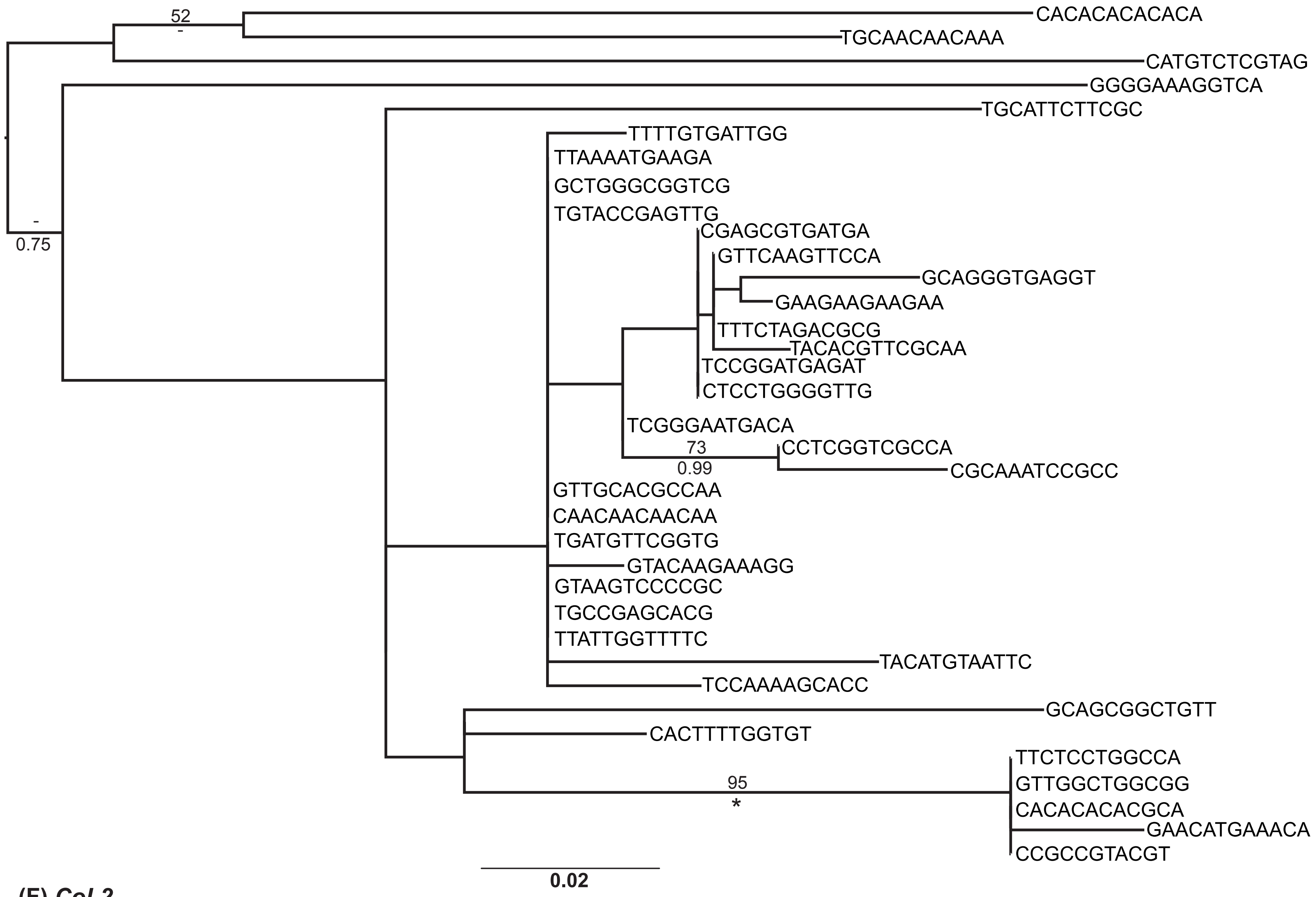
52
-
CACACACACACA
TGCAACAACAAA
CATGTCTCGTAG
GGGGAAAGGTCA
TGCATTCTTCGC
TTTTGTGATTGG
TTAAAATGAAGA
GCTGGGCGGTCG
TGTACCGAGTTG
-
0.75
CGAGCGTGATGA
GTTCAAGTTCCA
GCAGGGTGAGGT
GAAGAAGAAGAA
TTTCTAGACGCG
TACACGTTCGCAA
TCCGGATGAGAT
CTCCTGGGGTTG
TCGGGAATGACA
73
0.99
CCTCGGTCGCCA
CGCAAATCCGCC
GTTGCACGCCAA
CAACAACAACAA
TGATGTTCGGTG
GTACAAGAAAGG
GTAAGTCCCCGC
TGCCGAGCACG
TTATTGGTTTTC
TACATGTAATTC
TCCAAAAGCACC
GCAGCGGCTGTT
CACTTTTGGTGT
TTCTCCTGGCCA
GTTGGCTGGCGG
CACACACACGCA
95
*
GAACATGAAACA
CCGCCGTACGT
0.02

(F) ***CoL2***

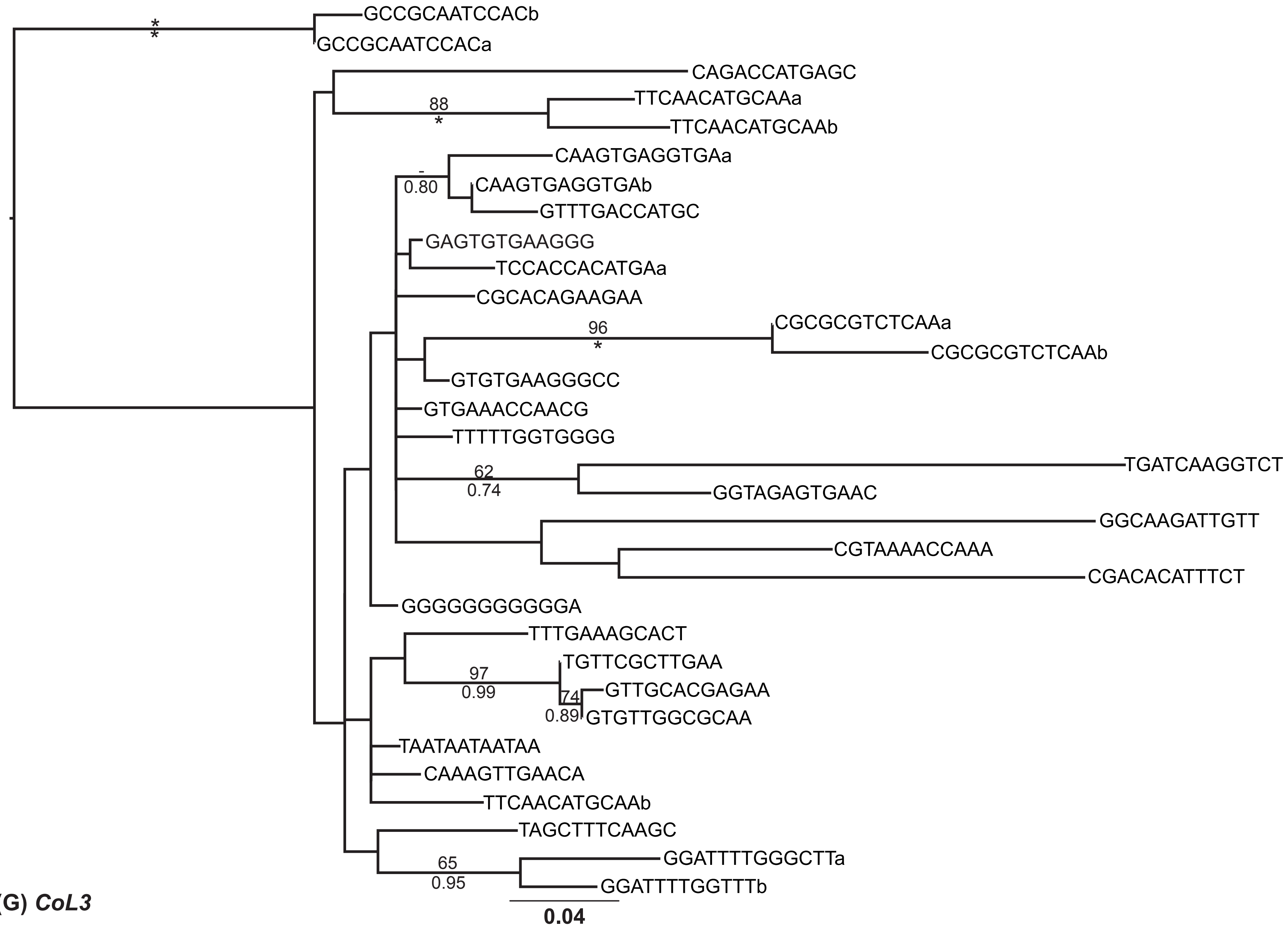
GCCGCAATCCACb
GCCGCAATCCACa
CAGACCATGAGC
88
TTCAACATGCAAa
TTCAACATGCAAb
CAAGTGAGGTGAa
0.80
CAAGTGAGGTGAb
GTTTGACCATGC
GAGTGTGAAGGG
TCCACCACATGAa
CGCACAGAAGAA
96
CGCGCGTCTCAAa
CGCGCGTCTCAAb
GTGTGAAGGGCC
GTGAAACCAACG
TTTTTGGTGGGG
62
0.74
TGATCAAGGTCT
GGTAGAGTGAAC
GGCAAGATTGTT
CGTAAAACCAAA
CGACACATTTCT
GGGGGGGGGGGA
TTTGAAAGCACT
97
0.99
TGTTCGCTTGAA
74
0.89
GTTGCACGAGAA
GTGTTGGCGCAA
TAATAATAATAA
CAAAGTTGAACA
TTCAACATGCAAb
TAGCTTTCAAGC
65
0.95
GGATTTTGGGCTTa
GGATTTTGGTTTb
0.04

(G) ***CoL3***

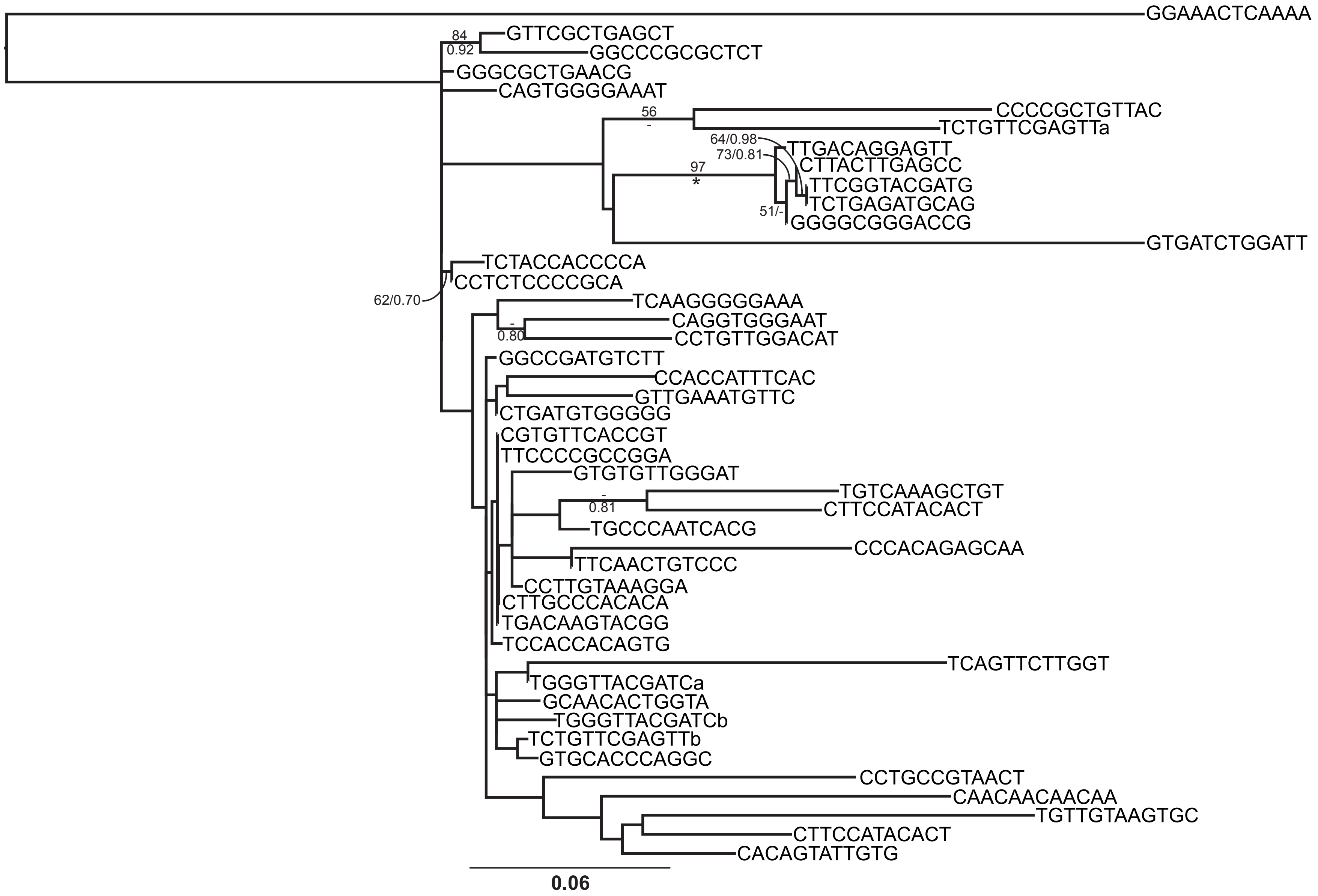

GGAAACTCAAAA
84
0.92
GTTCGCTGAGCT
GGCCCGCGCTCT
GGGCGCTGAACG
CAGTGGGGAAAT
56
-
CCCCGCTGTTAC
TCTGTTCGAGTTa
64/0.98
TTGACAGGAGTT
73/0.81
CTTACTTGAGCC
97
*
TTCGGTACGATG
TCTGAGATGCAG
51/-
GGGGCGGGACCG
GTGATCTGGATT
TCTACCACCCCA
CCTCTCCCCGCA
62/0.70
TCAAGGGGGAAA
-
0.80
CAGGTGGGAAT
CCTGTTGGACAT
GGCCGATGTCTT
CCACCATTTCAC
GTTGAAATGTTC
CTGATGTGGGGG
CGTGTTCACCGT
TTCCCCGCCGGA
GTGTGTTGGGAT
-
0.81
TGTCAAAGCTGT
CTTCCATACACT
TGCCCAATCACG
CCCACAGAGCAA
TTCAACTGTCCC
CCTTGTAAAGGA
CTTGCCCACACA
TGACAAGTACGG
TCCACCACAGTG
TCAGTTCTTGGT
TGGGTTACGATCa
GCAACACTGGTA
TGGGTTACGATCb
TCTGTTCGAGTTb
GTGCACCCAGGC
CCTGCCGTAACT
CAACAACAACAA
TGTTGTAAGTGC
CTTCCATACACT
CACAGTATTGTG
0.06

(H) ***CoL4***

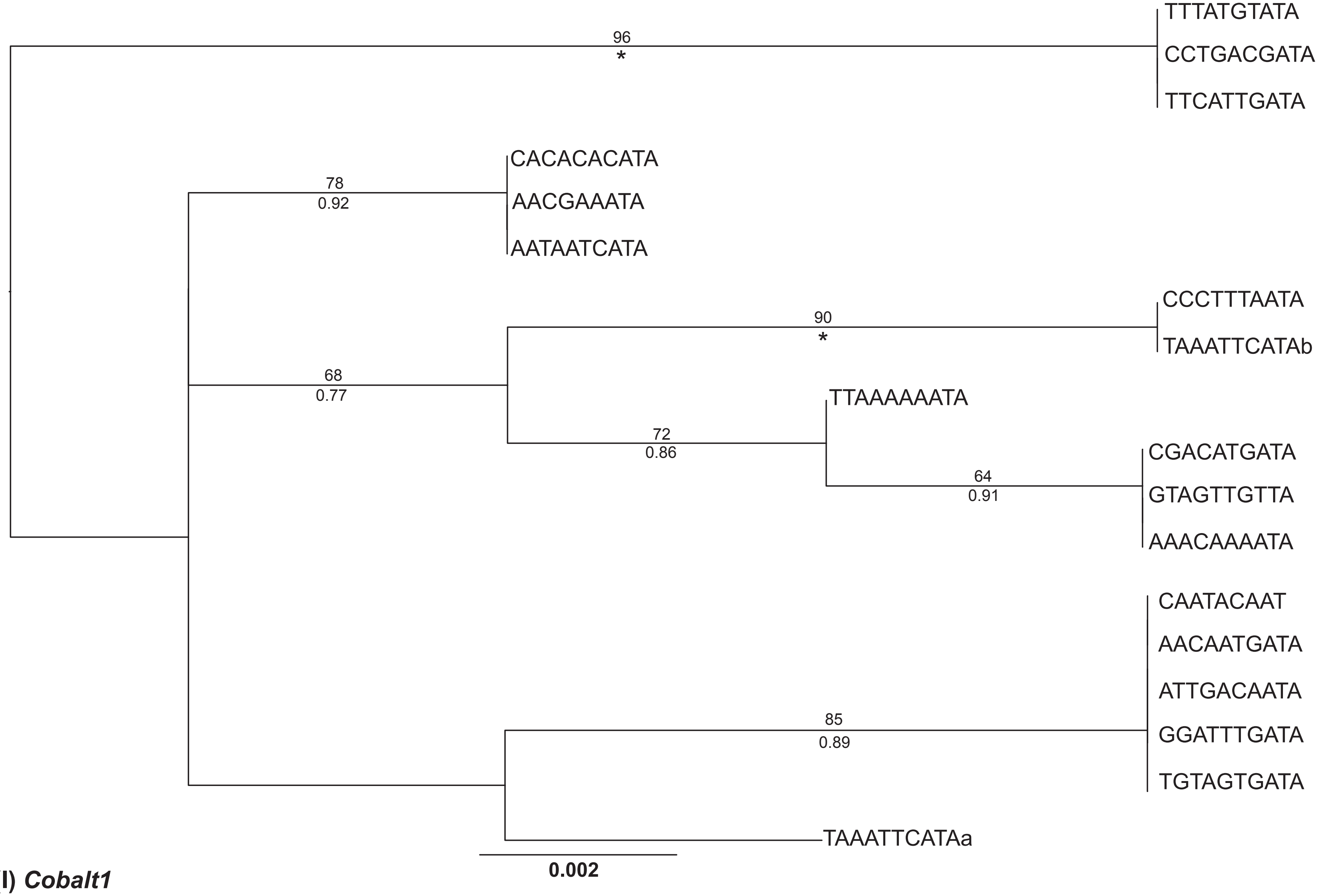

TTTATGTATA
CCTGACGATA
TTCATTGATA
96
*
CACACACATA
AACGAAATA
AATAATCATA
78
0.92
CCCTTTAATA
TAAATTCATAb
90
*
68
0.77
TTAAAAAATA
72
0.86
CGACATGATA
GTAGTTGTTA
AAACAAAATA
64
0.91
CAATACAAT
AACAATGATA
ATTGACAATA
GGATTTGATA
TGTAGTGATA
85
0.89
TAAATTCATAa
0.002

(I) *Cobalt1*

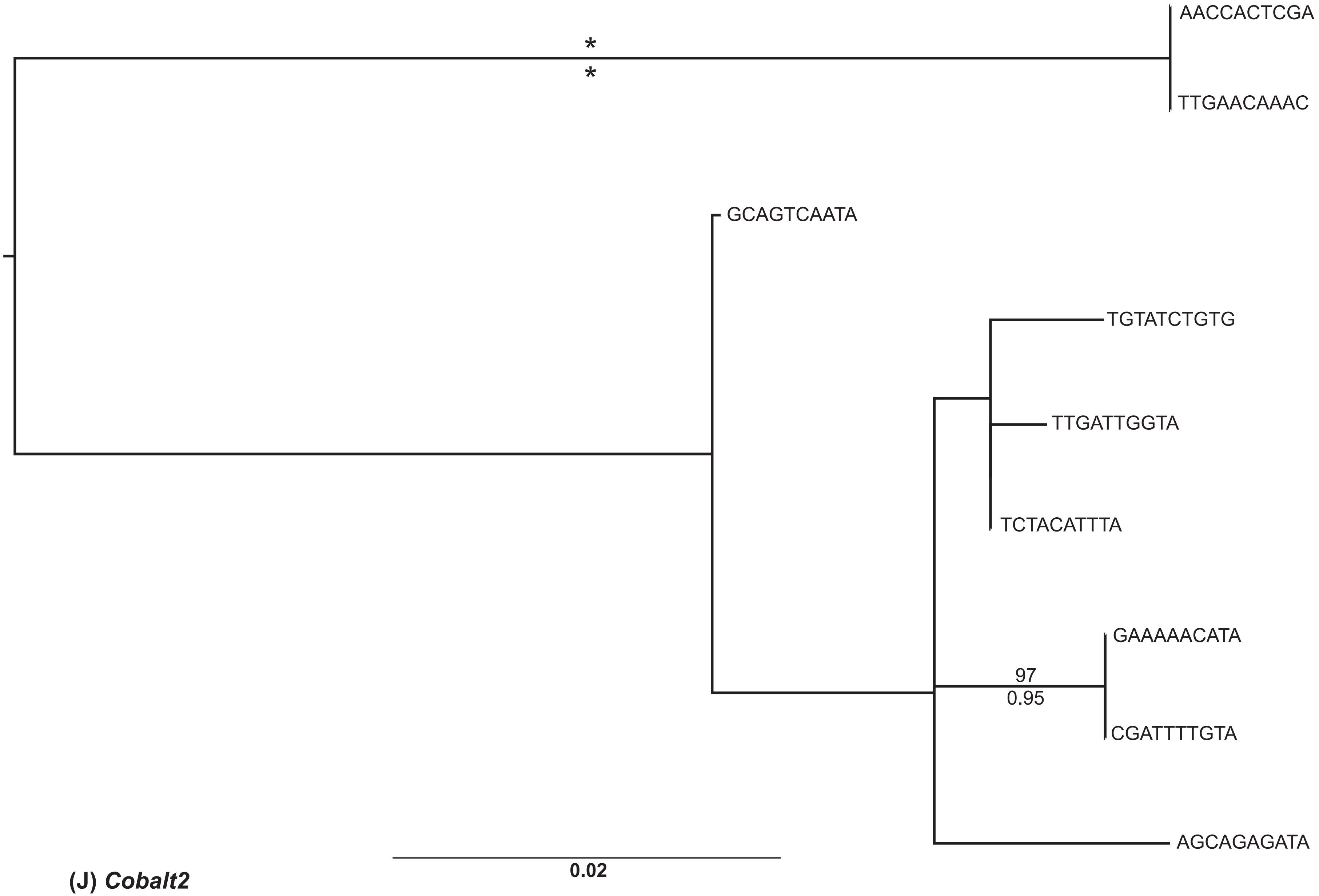
AACCACTCGA
TTGAACAAAC
*
*
GCAGTCAATA
TGTATCTGTG
TTGATTGGTA
TCTACATTTA
GAAAAACATA
97
0.95
CGATTTTGTA
AGCAGAGATA
0.02

(J) *Cobalt2*

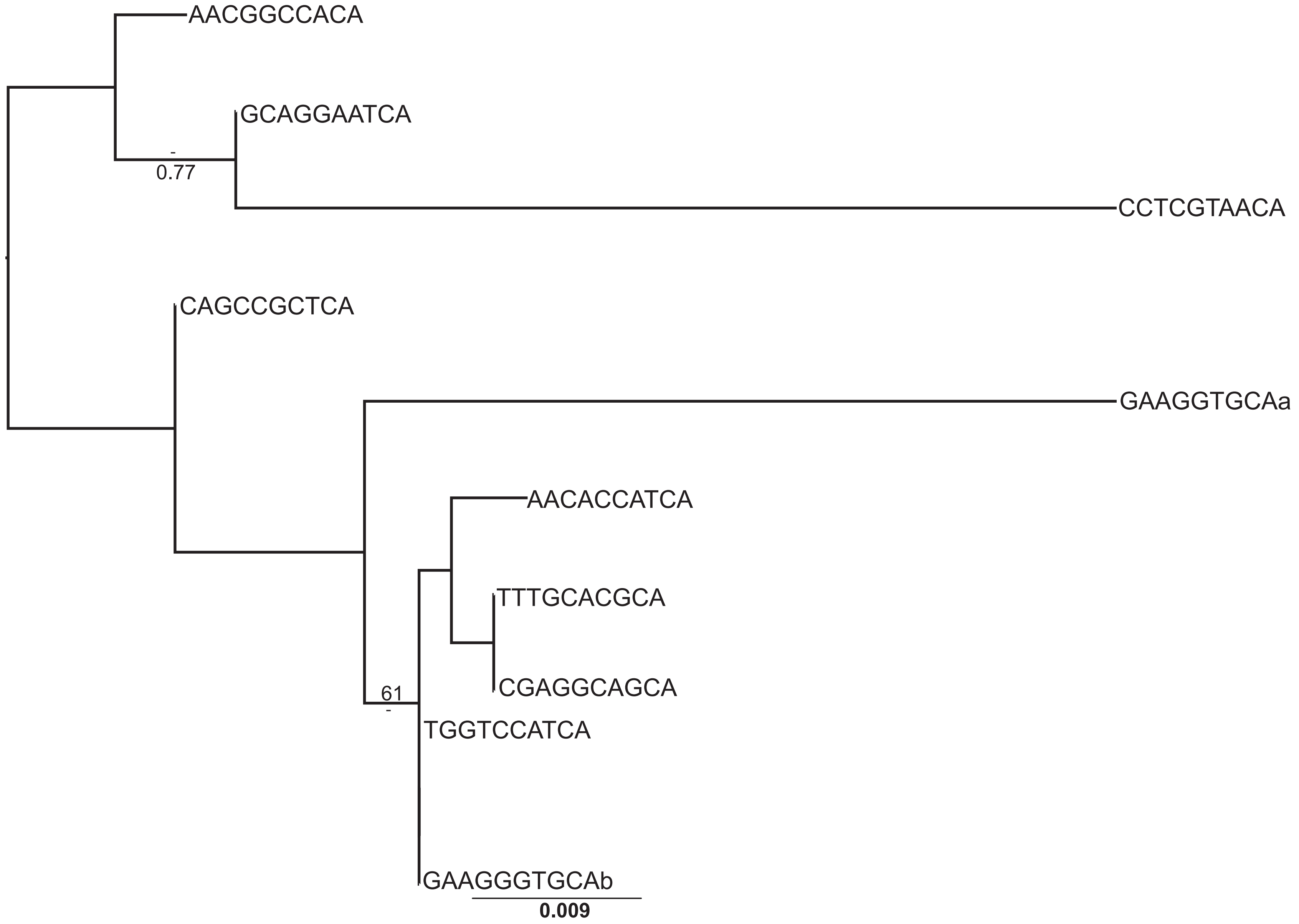
AACGGCCACA
GCAGGAATCA
-
0.77
CCTCGTAACA
CAGCCGCTCA
GAAGGTGCAa
AACACCATCA
TTTGCACGCA
CGAGGCAGCA
61
-
TGGTCCATCA
GAAGGGTGCAb
0.009

(K) ***CoCACTA1***

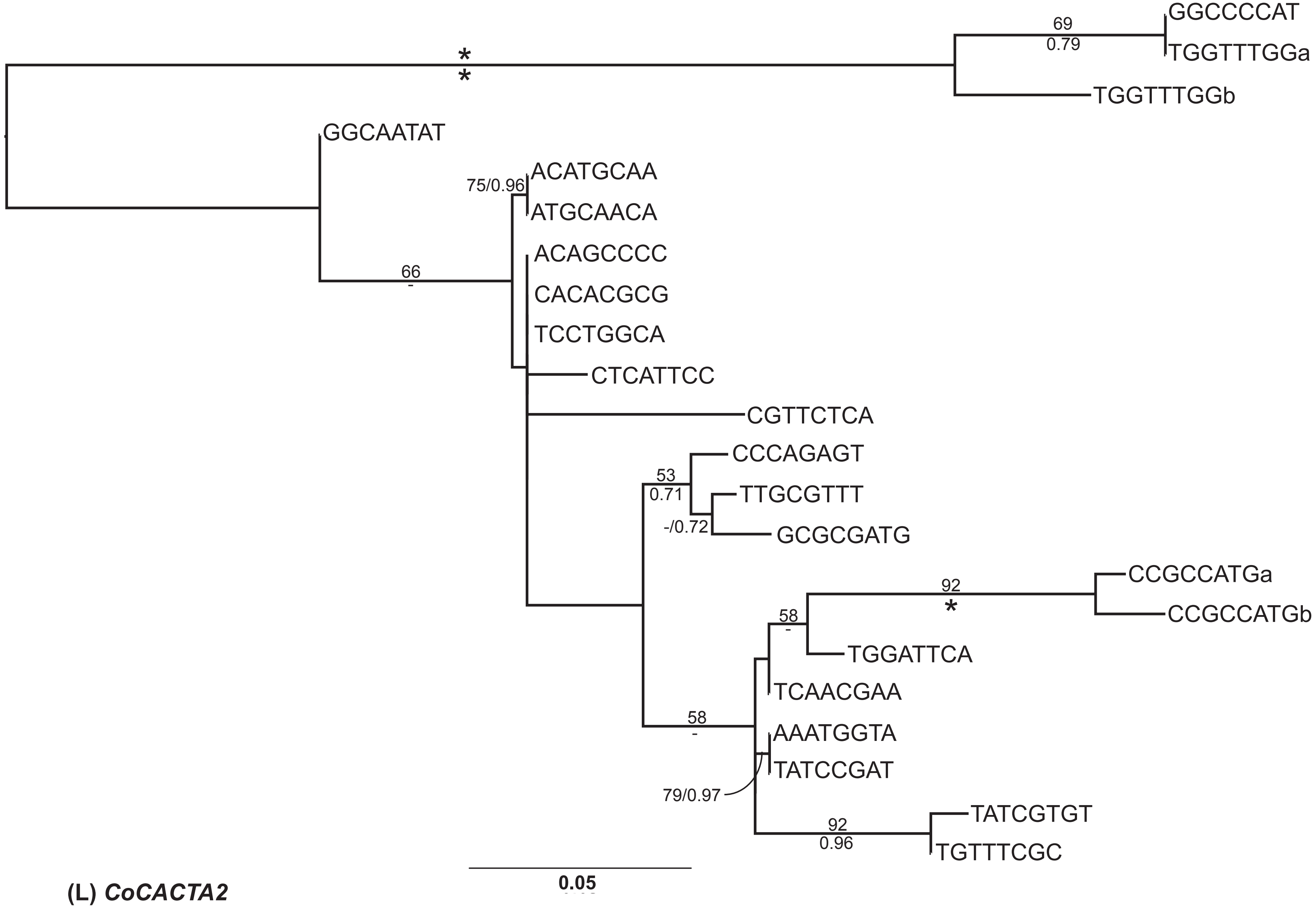
GGCCCCAT
TGGTTTGGa
TGGTTTGGb
69
0.79
*
*
GGCAATAT
75/0.96
ACATGCAA
ATGCAACA
66
-
ACAGCCCC
CACACGCG
TCCTGGCA
CTCATTCC
CGTTCTCA
53
0.71
CCCAGAGT
TTGCGTTT
-/0.72
GCGCGATG
92
*
CCGCCATGa
CCGCCATGb
58
-
TGGATTCA
TCAACGAA
58
-
AAATGGTA
TATCCGAT
79/0.97
92
0.96
TATCGTGT
TGTTTCGC
0.05

(L) ***CoCACTA2***

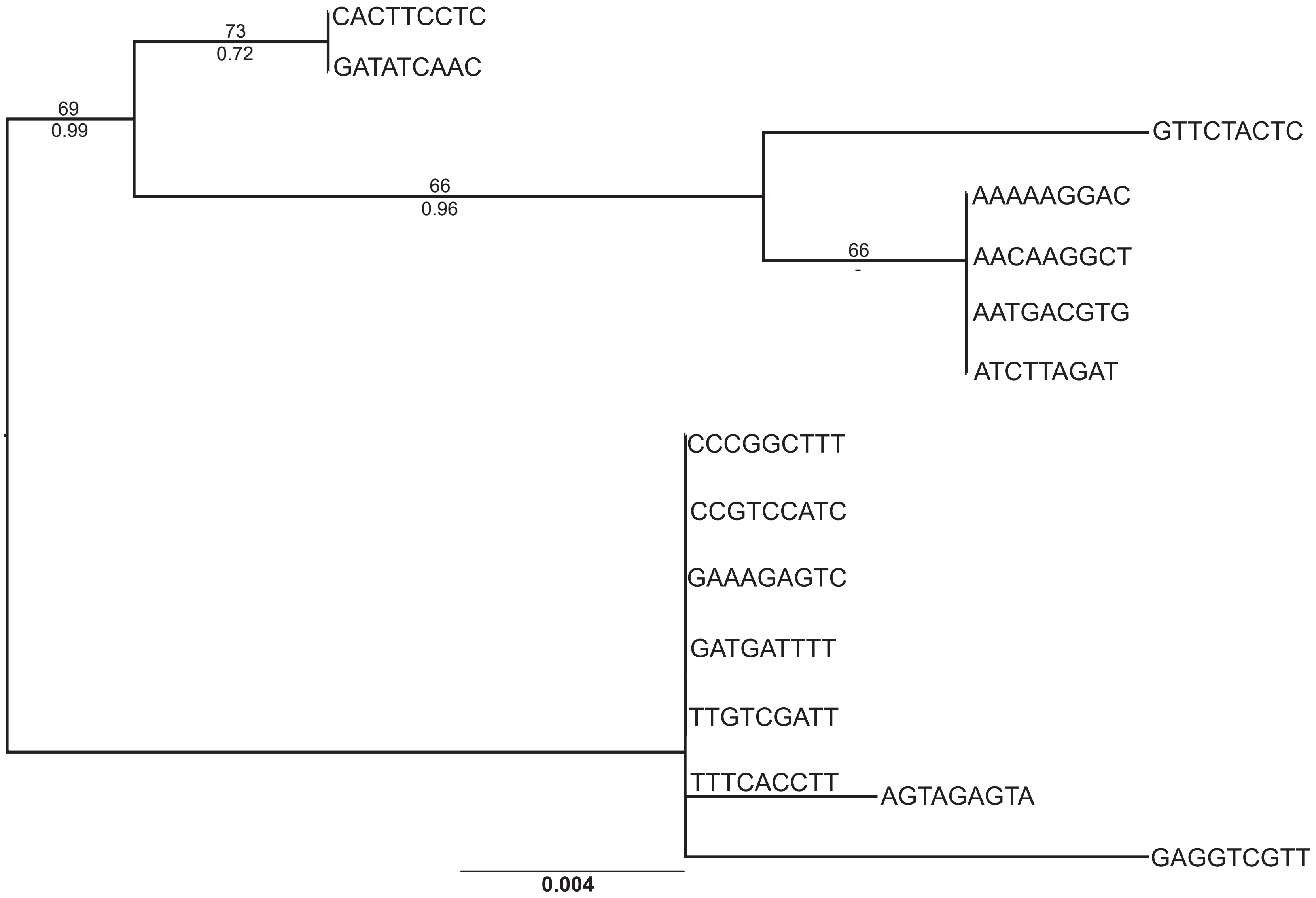
CACTTCCTC
GATATCAAC
73
0.72
69
0.99
GTTCTACTC
66
0.96
AAAAAGGAC
AACAAGGCT
66
-
AATGACGTG
ATCTTAGAT
CCCGGCTTT
CCGTCCATC
GAAAGAGTC
GATGATTTT
TTGTCGATT
TTTCACCTT
AGTAGAGTA
GAGGTCGTT
0.004

(M) ***Com1***

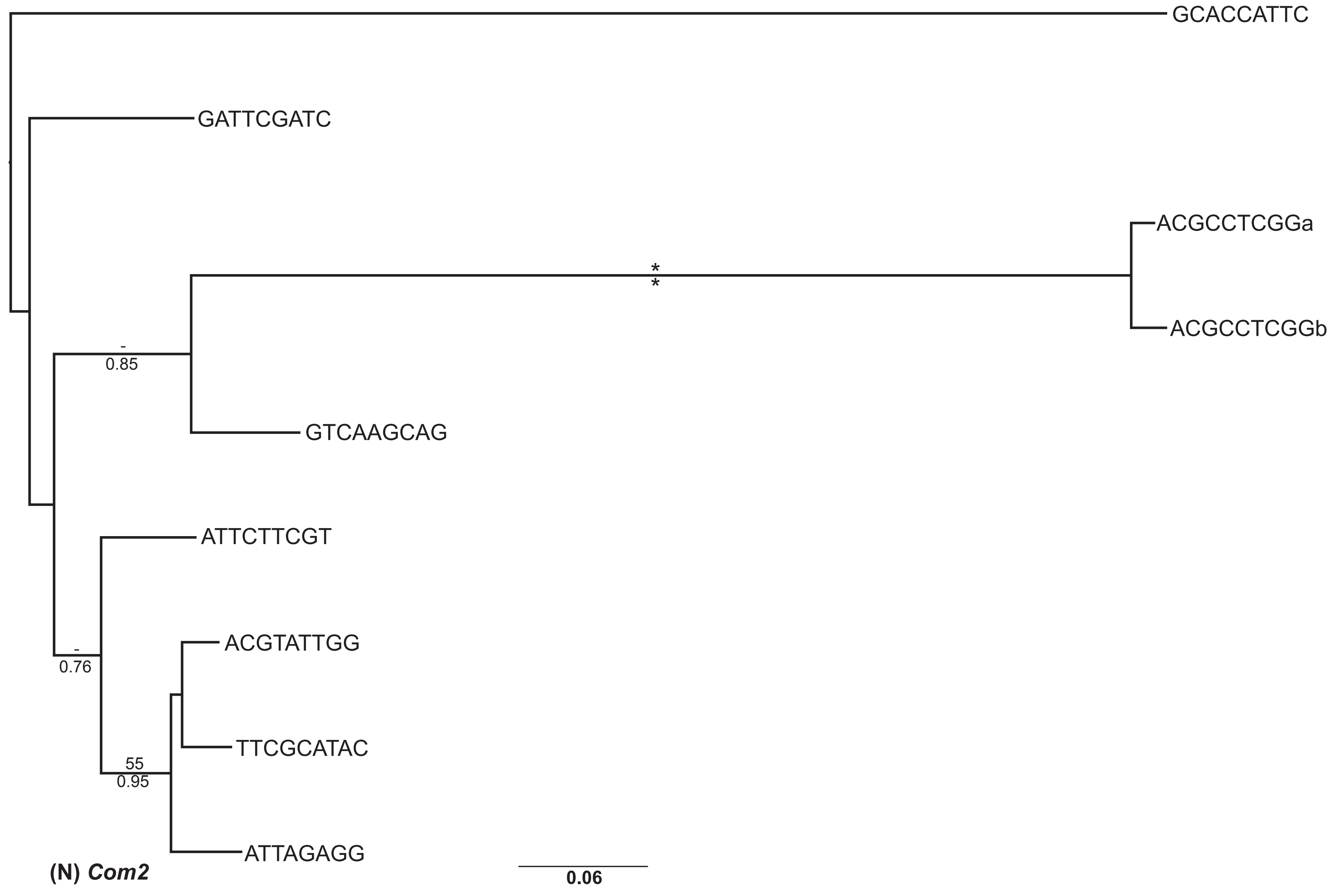
GCACCATTC
GATTCGATC
ACGCCTCGGa
*
*
ACGCCTCGGb
-
0.85
GTCAAGCAG
ATTCTTCGT
-
0.76
ACGTATTGG
TTCGCATAC
55
0.95
ATTAGAGG
0.06
(N) ***Com2***

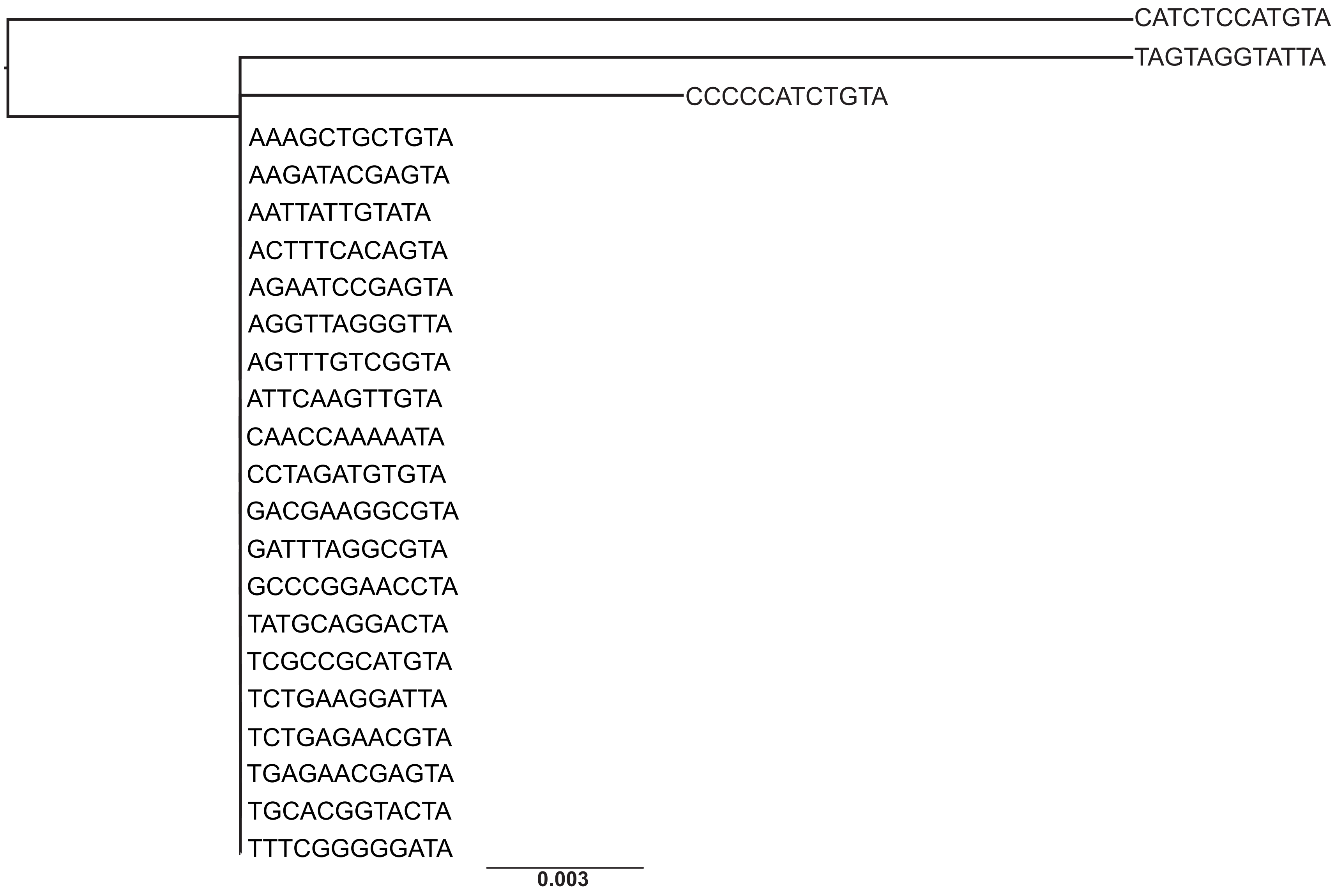
CATCTCCATGTA
TAGTAGGTATTA
CCCCCATCTGTA
AAAGCTGCTGTA
AAGATACGAGTA
AATTATTGTATA
ACTTTCACAGTA
AGAATCCGAGTA
AGGTTAGGGTTA
AGTTTGTCGGTA
ATTCAAGTTGTA
CAACCAAAAATA
CCTAGATGTGTA
GACGAAGGCGTA
GATTTAGGCGTA
GCCCGGAACCTA
TATGCAGGACTA
TCGCCGCATGTA
TCTGAAGGATTA
TCTGAGAACGTA
TGAGAACGAGTA
TGCACGGTACTA
TTTCGGGGGATA
0.003

(O) ***Cop1***

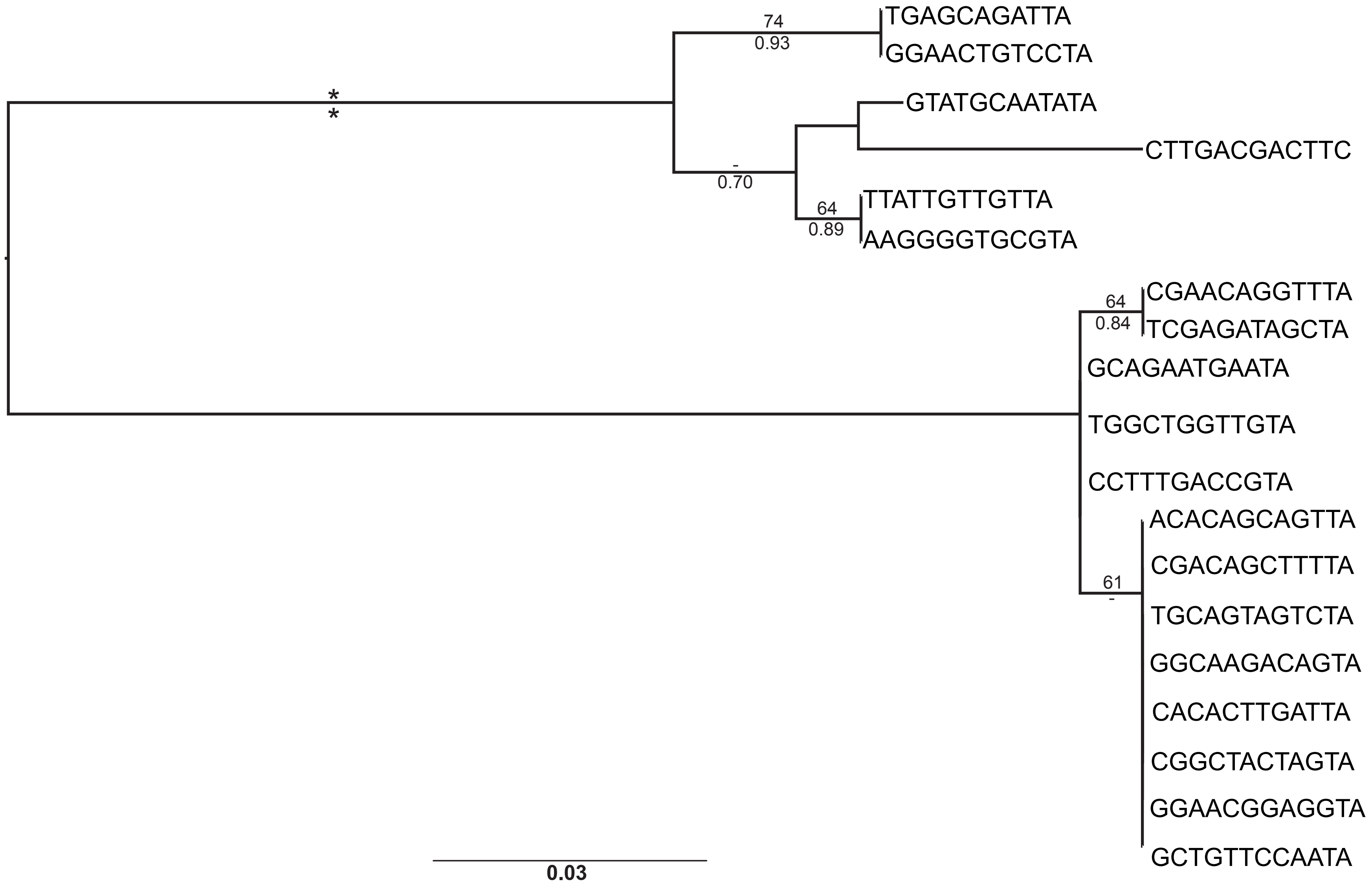
74
0.93
TGAGCAGATTA
GGAACTGTCCTA
*
*
GTATGCAATATA
CTTGACGACTTC
-
0.70
64
0.89
TTATTGTTGTTA
AAGGGGTGCGTA
64
0.84
CGAACAGGTTTA
TCGAGATAGCTA
GCAGAATGAATA
TGGCTGGTTGTA
CCTTTGACCGTA
ACACAGCAGTTA
CGACAGCTTTTA
61
-
TGCAGTAGTCTA
GGCAAGACAGTA
CACACTTGATTA
CGGCTACTAGTA
GGAACGGAGGTA
GCTGTTCCAATA
0.03

(P) ***Cop2***

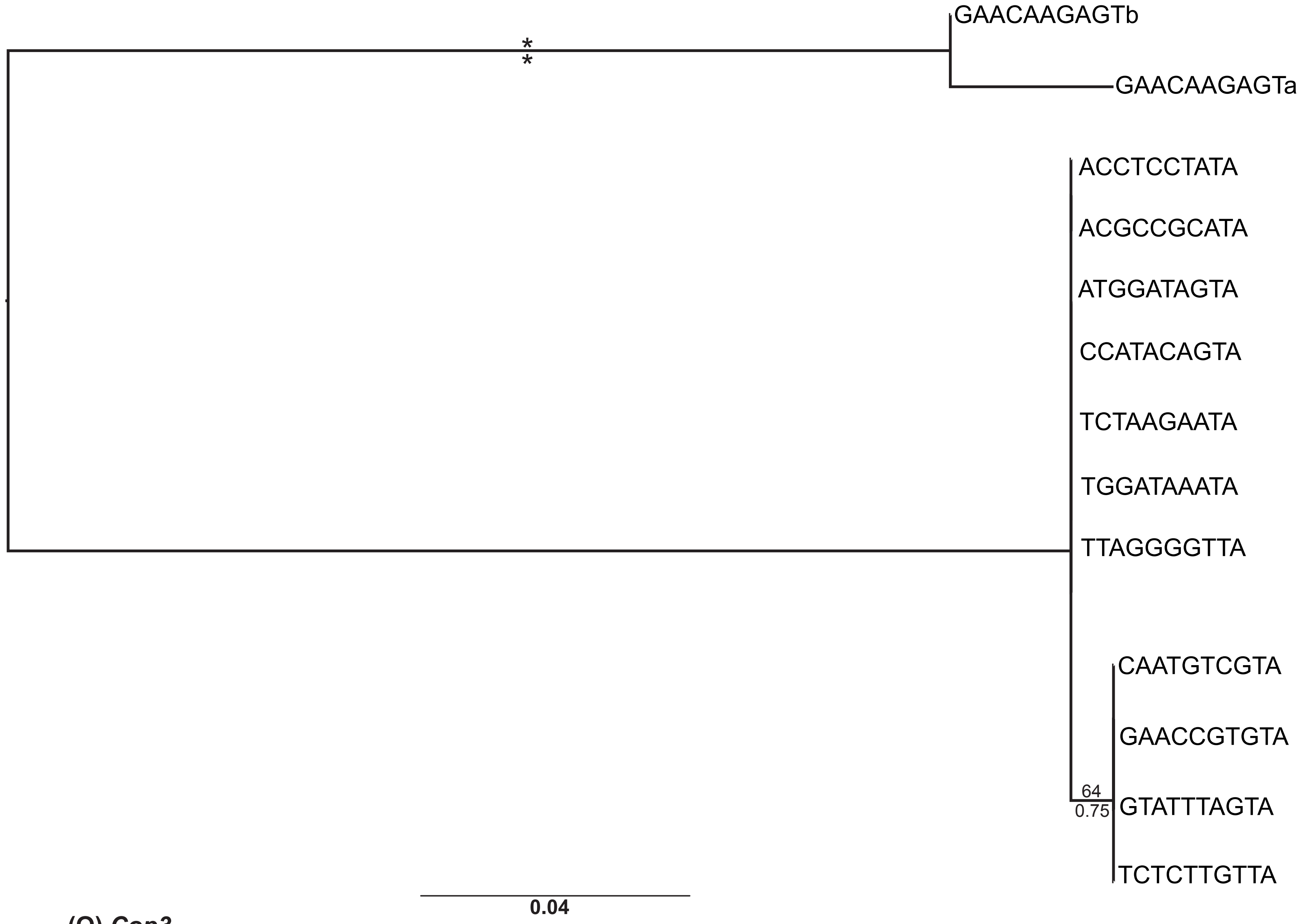

GAACAAGAGTb
*
*
GAACAAGAGTa
ACCTCCTATA
ACGCCGCATA
ATGGATAGTA
CCATACAGTA
TCTAAGAATA
TGGATAAATA
TTAGGGGTTA
CAATGTCGTA
GAACCGTGTA
64
0.75
GTATTTAGTA
TCTCTTGTTA
0.04

(Q) ***Cop3***

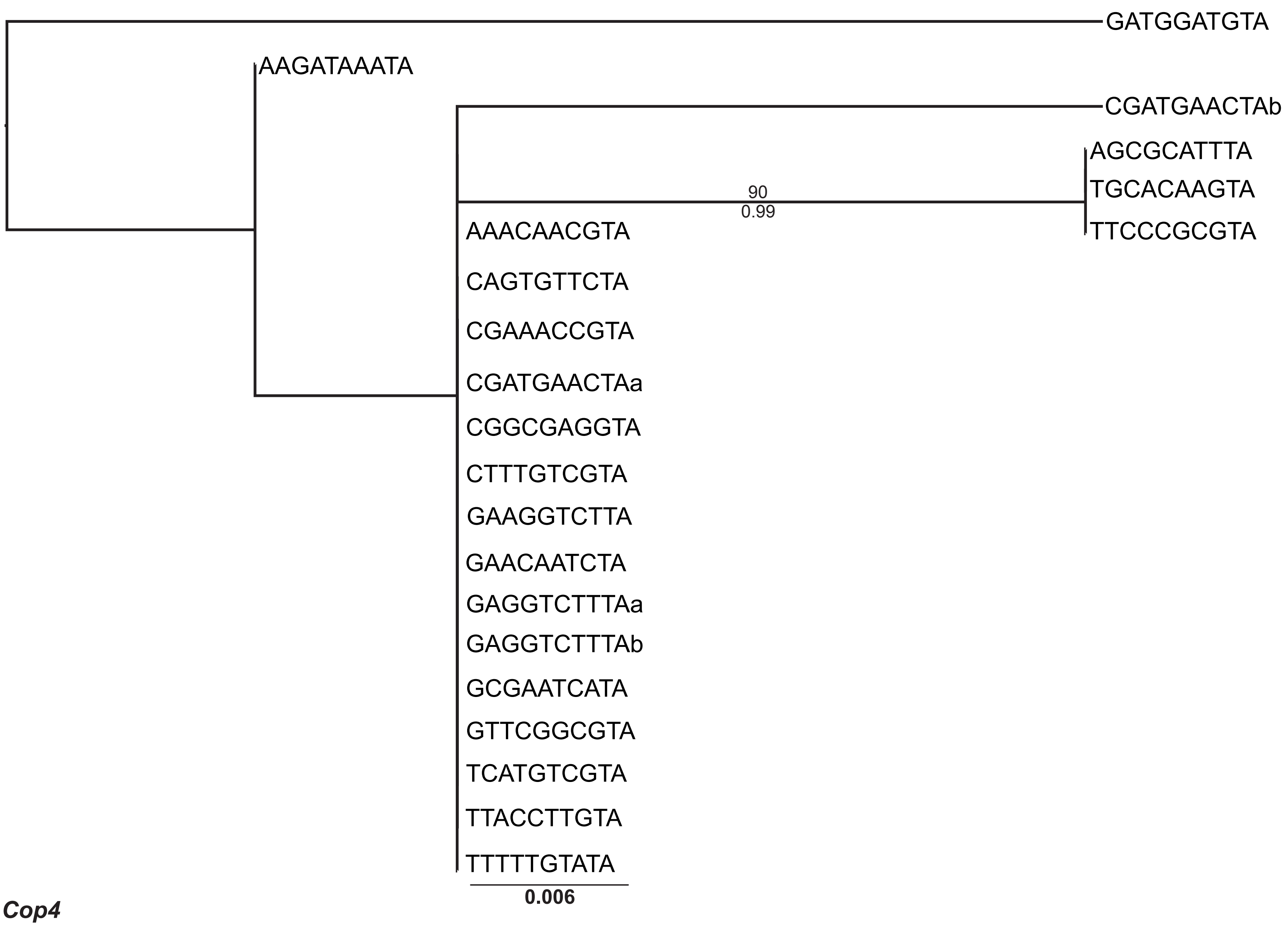
GATGGATGTA
AAGATAAATA
CGATGAACTAb
AGCGCATTTA
TGCACAAGTA
TTCCCGCGTA
90
0.99
AAACAACGTA
CAGTGTTCTA
CGAAACCGTA
CGATGAACTAa
CGGCGAGGTA
CTTTGTCGTA
GAAGGTCTTA
GAACAATCTA
GAGGTCTTTAa
GAGGTCTTTAb
GCGAATCATA
GTTCGGCGTA
TCATGTCGTA
TTACCTTGTA
TTTTTGTATA
0.006

(R) ***Cop4***

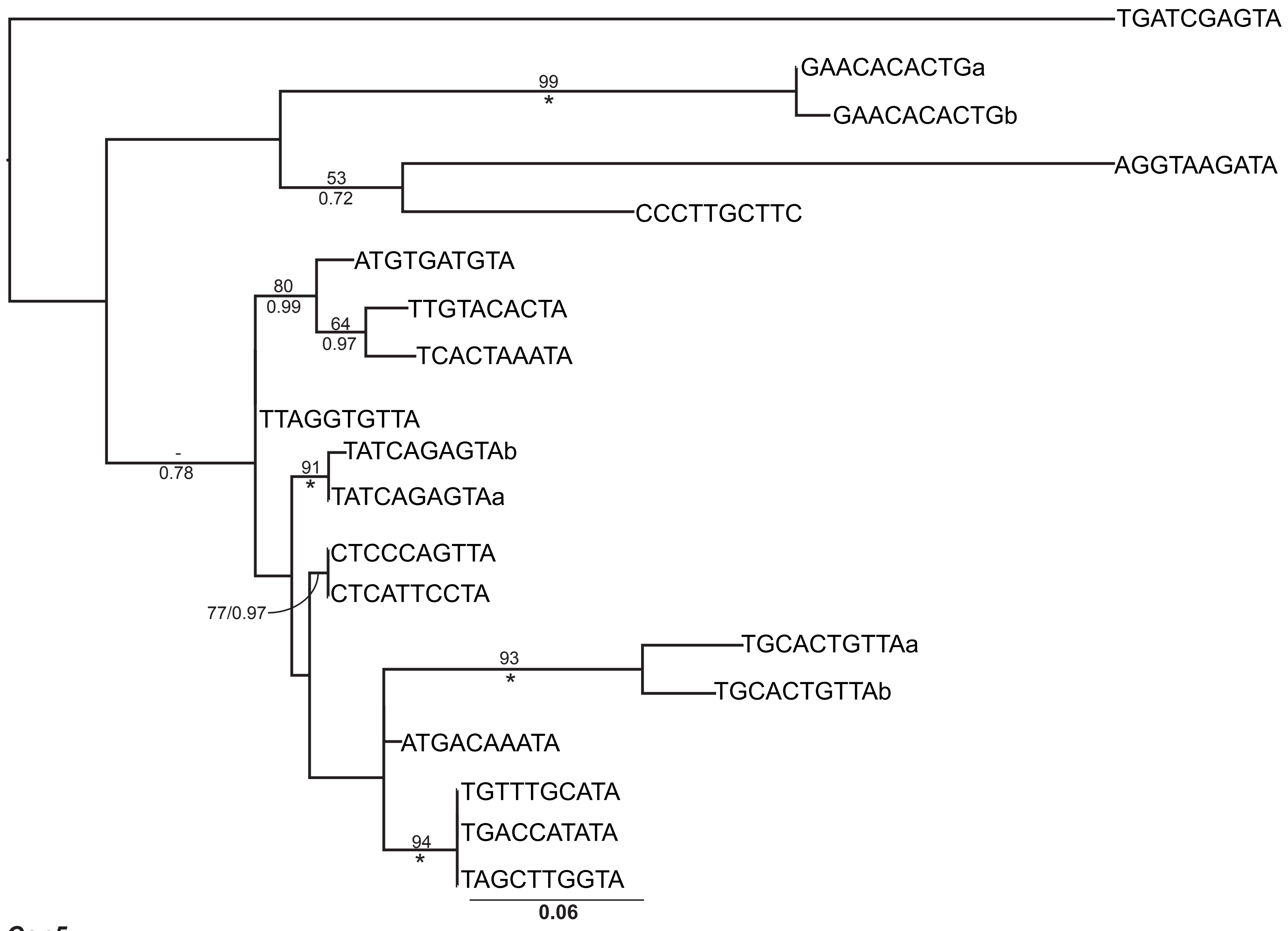
TGATCGAGTA
99
*
GAACACACTGa
GAACACACTGb
53
0.72
AGGTAAGATA
CCCTTGCTTC
80
0.99
ATGTGATGTA
64
0.97
TTGTACACTA
TCACTAAATA
TTAGGTGTTA
-
0.78
91
*
TATCAGAGTAb
TATCAGAGTAa
CTCCCAGTTA
CTCATTCCTA
77/0.97
93
*
TGCACTGTTAa
TGCACTGTTAb
ATGACAAATA
94
*
TGTTTGCATA
TGACCATATA
TAGCTTGGTA
0.06

(S) ***Cop5***

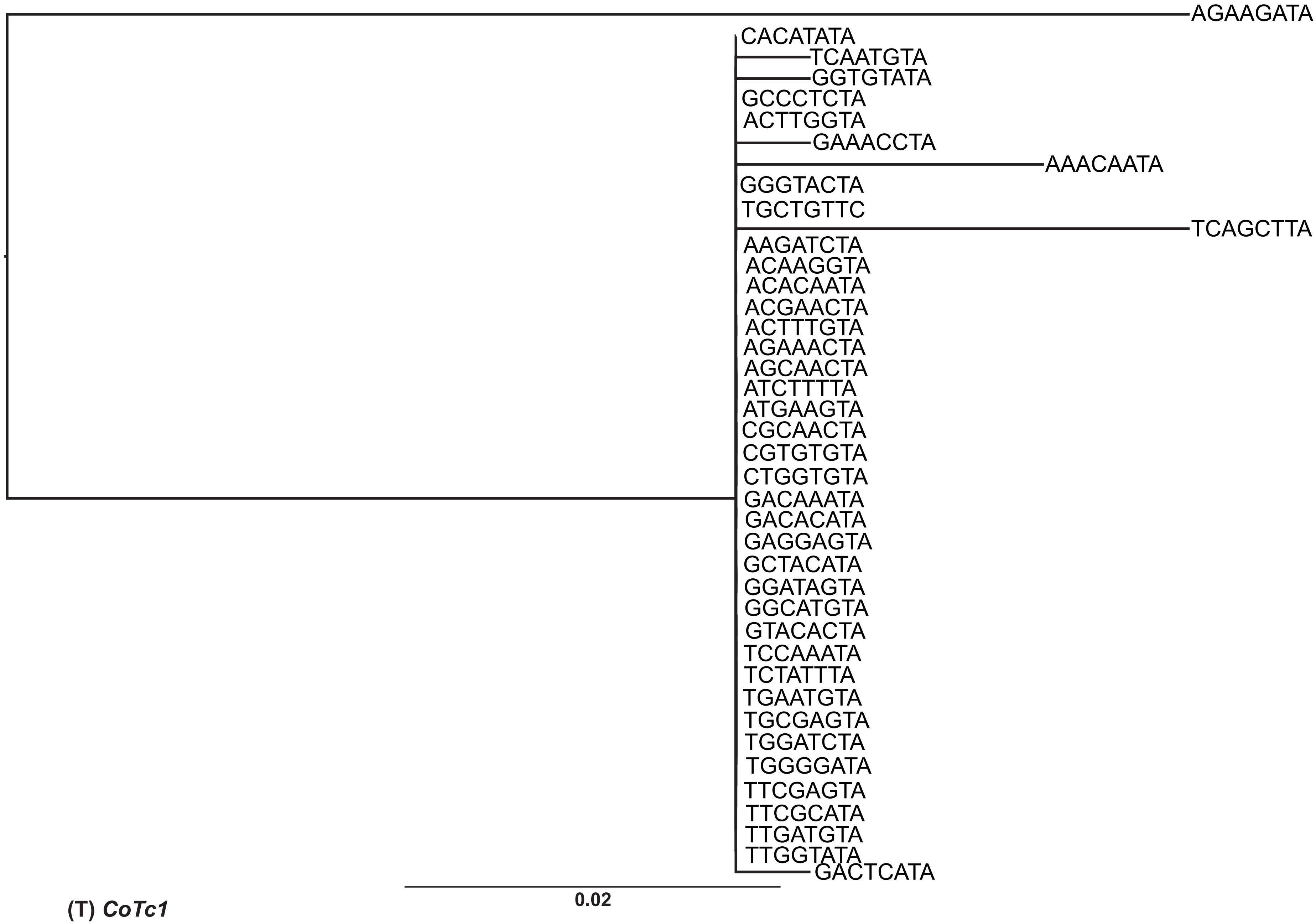
AGAAGATA
CACATATA
TCAATGTA
GGTGTATA
GCCCTCTA
ACTTGGTA
GAAACCTA
AAACAATA
GGGTACTA
TGCTGTTC
TCAGCTTA
AAGATCTA
ACAAGGTA
ACACAATA
ACGAACTA
ACTTTGTA
AGAAACTA
AGCAACTA
ATCTTTTA
ATGAAGTA
CGCAACTA
CGTGTGTA
CTGGTGTA
GACAAATA
GACACATA
GAGGAGTA
GCTACATA
GGATAGTA
GGCATGTA
GTACACTA
TCCAAATA
TCTATTTA
TGAATGTA
TGCGAGTA
TGGATCTA
TGGGGATA
TTCGAGTA
TTCGCATA
TTGATGTA
TTGGTATA
GACTCATA
0.02

(T) ***CoTc1***

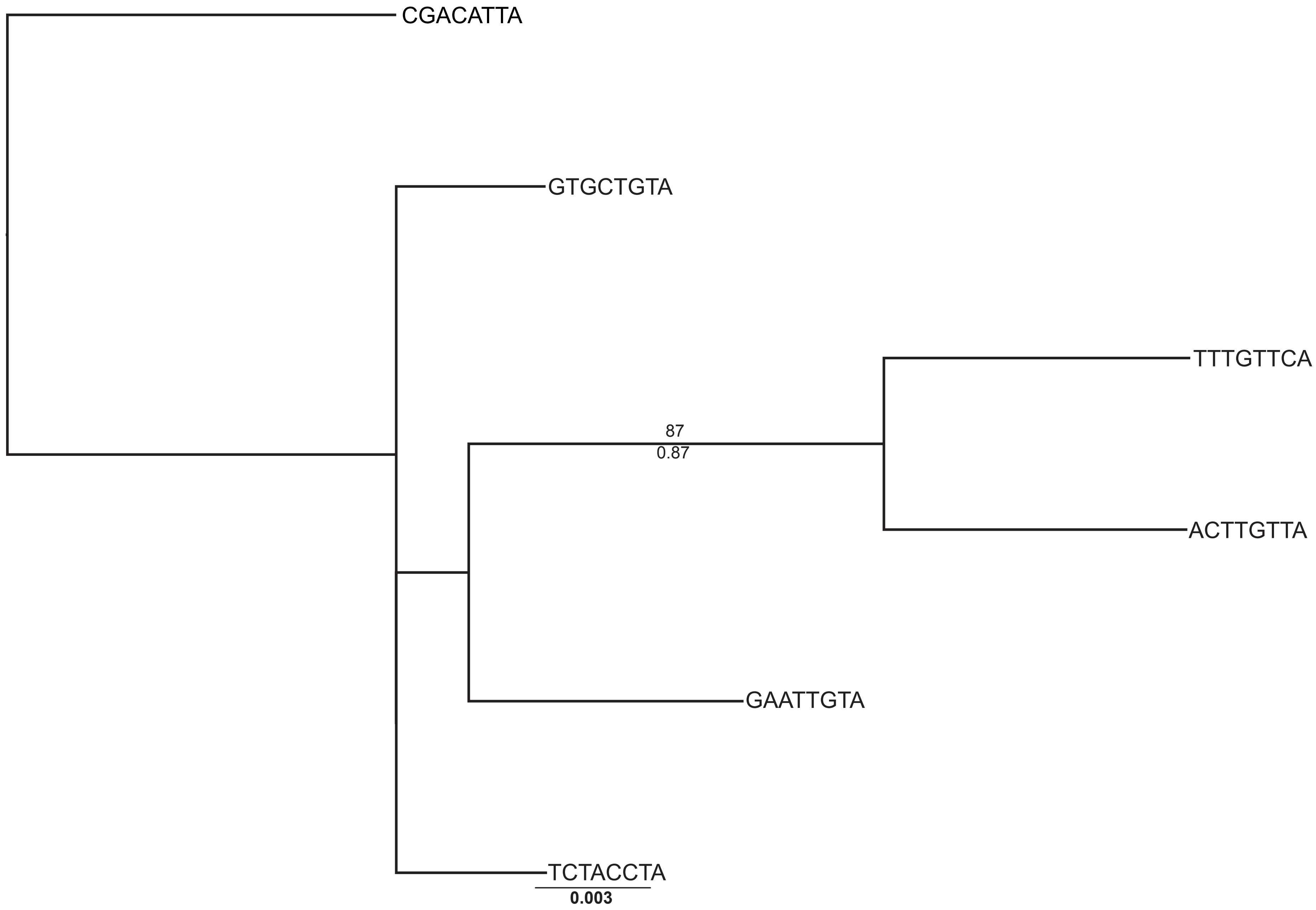
CGACATTA
GTGCTGTA
TTTGTTCA
87
0.87
ACTTGTTA
GAATTGTA
TCTACCTA
0.003

(U) *CoTc2*
