# Supplementary figures and images for: The Holozoan Capsaspora owczarzaki Possesses a Diverse Complement of Active Transposable Element Families
Source: Genome Biol Evol. 2014 Apr 2;6(4):949–63. doi: 10.1093/gbe/evu068 (PMC4007536; doi:10.1093/gbe/evu068)

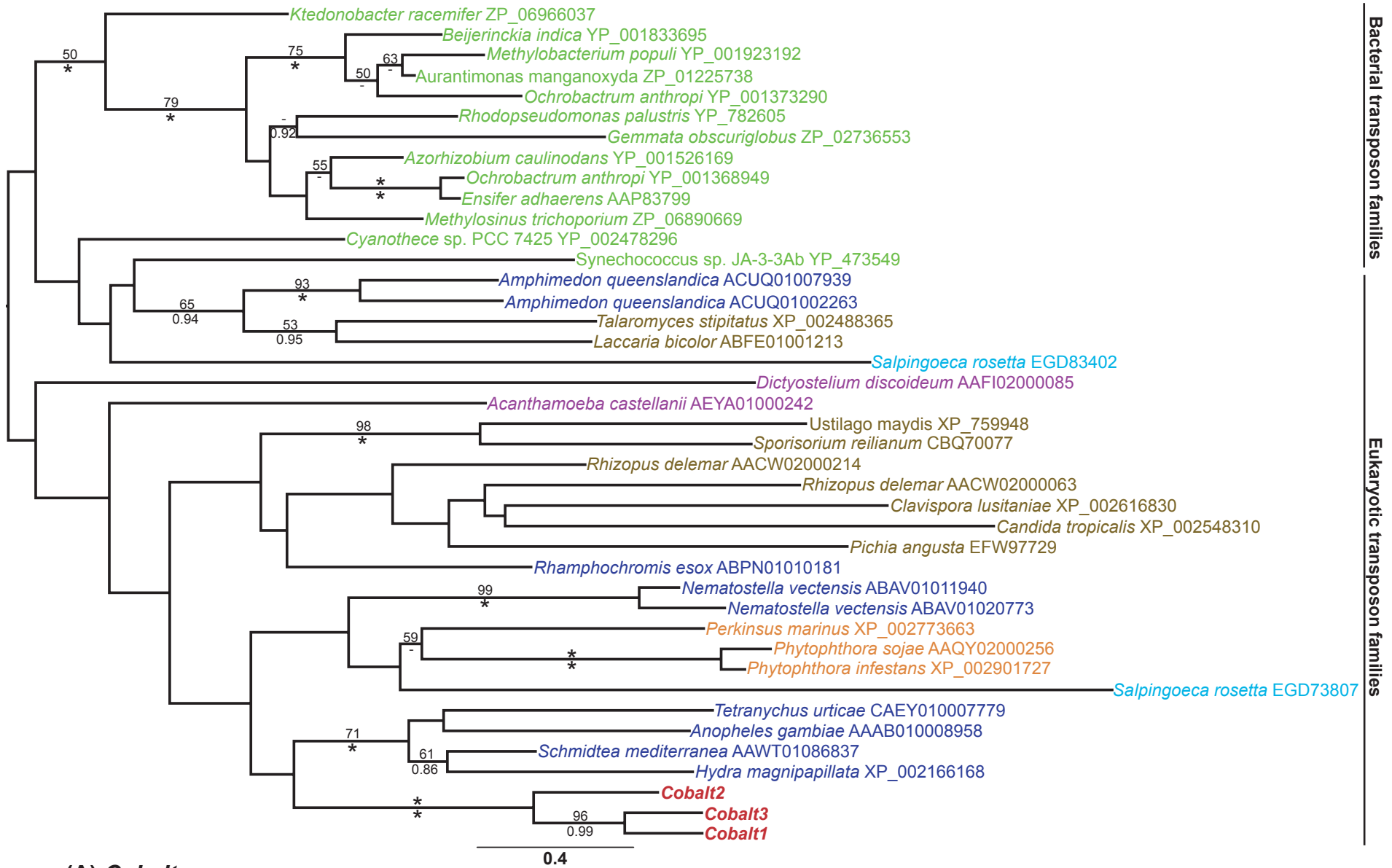

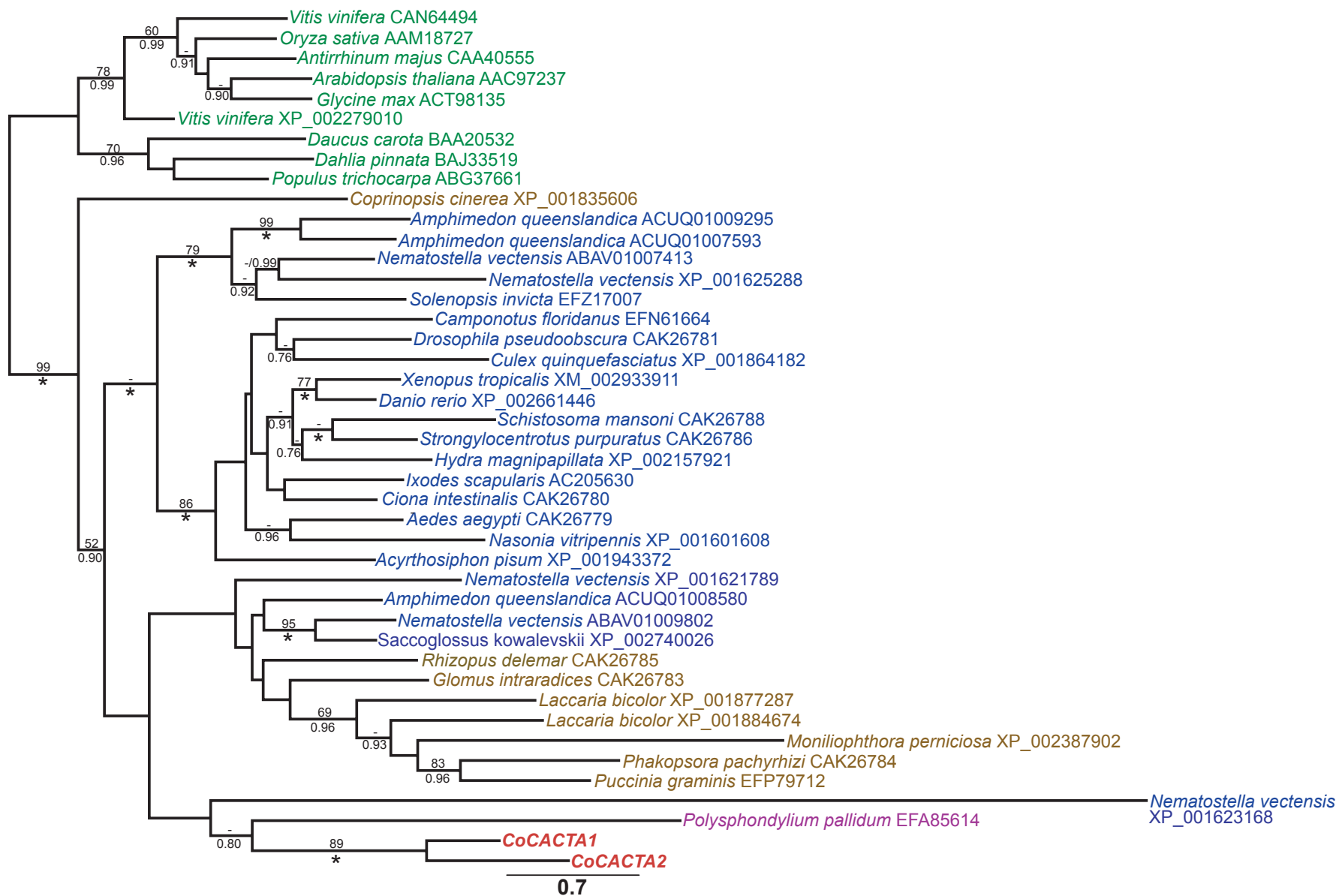

Plant CACTA Families

Opisthokont CACTA Families

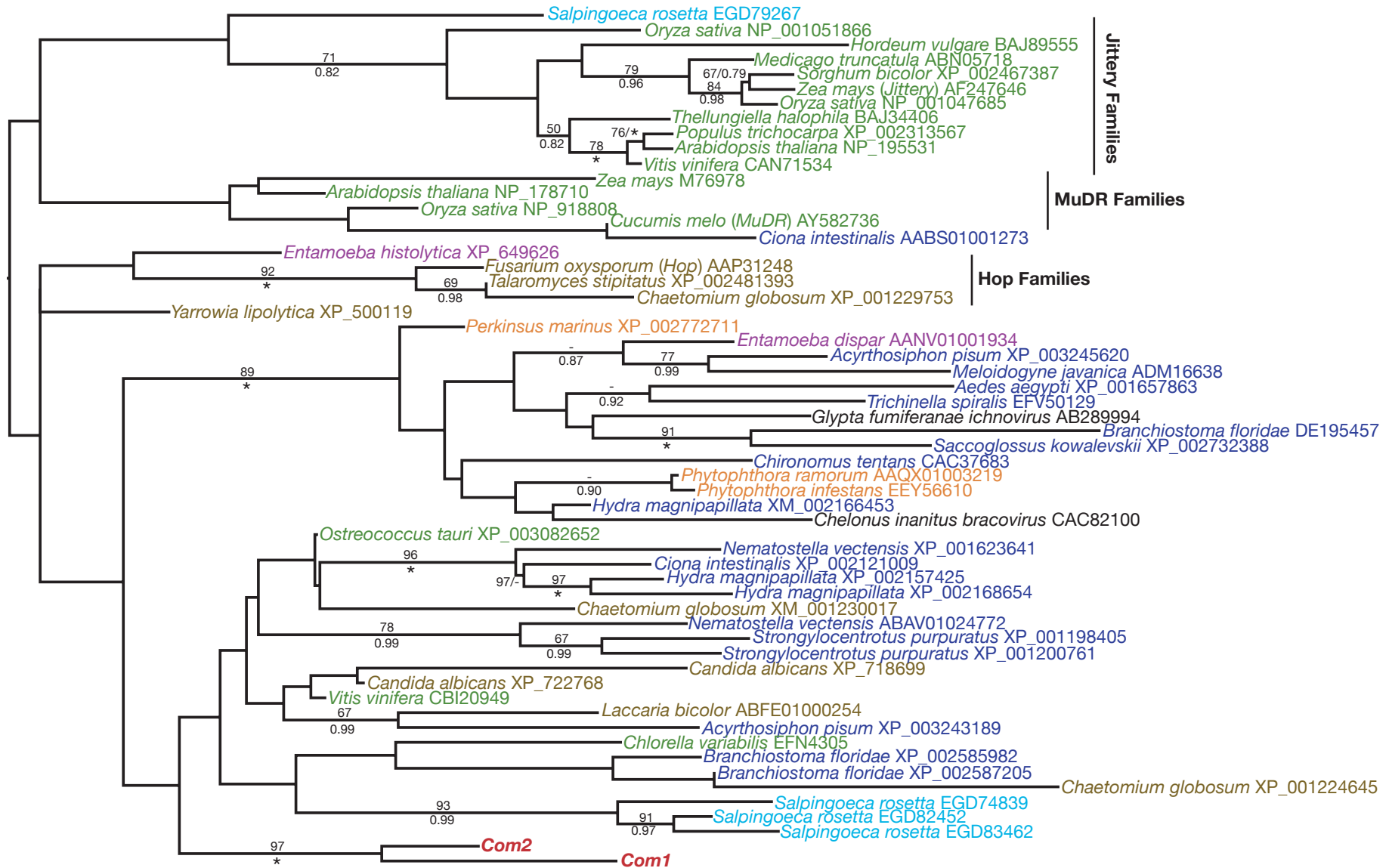

(C) MULE

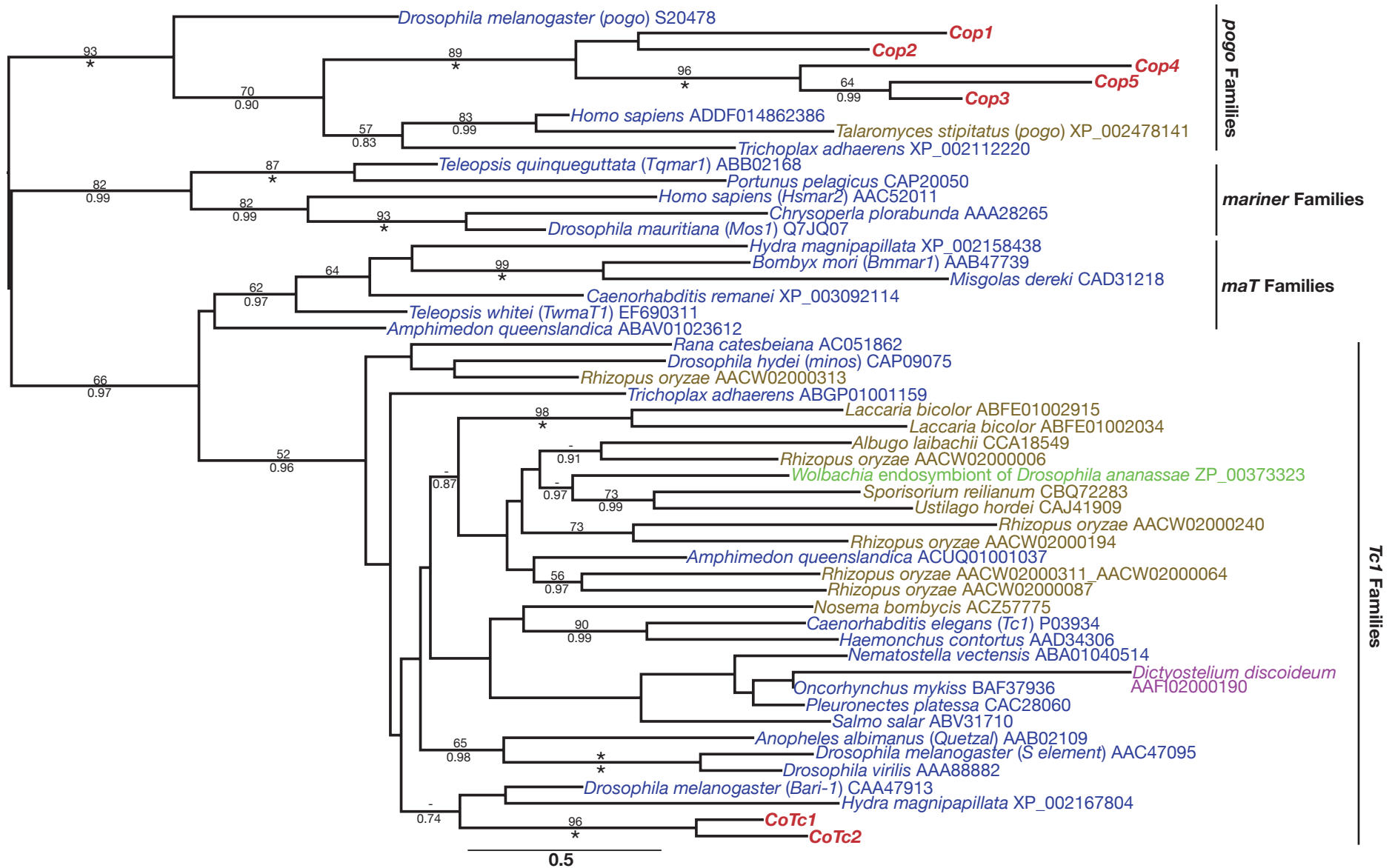

(D) Tc1

Supplement: Supplementary Data [file supp_evu068_suppl_data.zip › Figure S1.pdf]

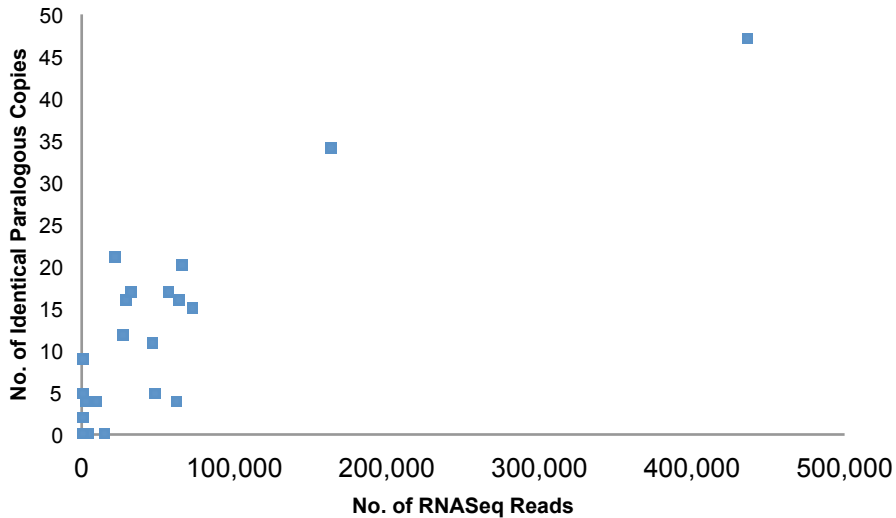

Supplement: Supplementary Data [file supp_evu068_suppl_data.zip › Figure S3.pdf]
