# Supplementary material for: The Holozoan Capsaspora owczarzaki Possesses a Diverse Complement of Active Transposable Element Families
Source: Genome Biol Evol. 2014 Apr 2;6(4):949–63. doi: 10.1093/gbe/evu068 (PMC4007536; doi:10.1093/gbe/evu068)
Supplement: Supplementary Data [file supp_evu068_suppl_data.zip › Supplementary Information File 1.doc]

Supplementary Information

**The holozoan *Capsaspora owczarzaki* possesses a diverse complement of active transposable element families**

**Supplementary Information Files 1-4**

**Figures S1-S4**

**Supplementary Information File 2**

Normalized expression levels of the 23 transposable element families in three stages in the life cycle of *C. owczarzaki*. Three sets of replicates were generated for each stage. Differences in expression levels between stages were tested with analyses of variance (ANOVA); in cases where significant differences across stages were observed, the highest two stages were compared using a *t*-test. Significance levels: *<0.05, **<0.01, ***<0.001.

**Supplementary Information File 3**

Phylip alignments used in all analyses used to generate phylogenies and nucleotide diversity indices for the 22 multicopy transposable element families.

**Supplementary Information File 4**

Phylip alignments used in all analyses used to generate the maximum likelihood and the Bayesian Inference phylogenies for the seven superfamilies identified in the *C. owczarzaki* genome.

**Figure Legends**

**Figure S1.** Maximum likelihood Tnpase phylogenies of (A) *Cobalt*, (B) *CACTA*, (C) *MULE* and (D) *Tc1* families. The phylogenies were constructed from (A) 165, (B), 300 (C) 159 and (D) 204 aligned amino acid positions using the PROTCAT model with the LG substitution matrix. Bacterial proteins are written in light green font and viral proteins are written in black font, the phylogeny is otherwise formatted in the style of Figures 1-3.

**Figure S2**. Base composition of target site motifs for transposable element families in the genome of *C. owczarzaki*. The height of each nucleotide is proportional to its frequency within the family target sites.

**Figure S3**. Scatter chart showing the relationship between transposable element family expression levels and the number of identical paralogous copies within the family. The data point for each transposable element family is represented by a single blue square

**Figure S4.** Maximum likelihood phylogenies of termini sequences from (A) *Cocv2*, (B) *Cocv3*, (C) *Cocv4*, (D) *Cocv5*, (E) *CoL1*, (F) *CoL2*, (G) *CoL3*, (H) *CoL4*, (I) *Cobalt1*, (J) *Cobalt2*, (K) *CoCACTA1*, (L) *CoCACTA2*, (M) *Com1*, (N) *Com2*, (O) *Cop1*, (P) *Cop2*, (Q) *Cop3*, (R) *Cop4*, (S) *Cop5*, (T) *CoTc1* and (U) *CoTc2*. The phylogenies were constructed from (A) 182, (B) 195, (C) 90, (D) 316, (E) 152, (F) 144, (G) 159, (H) 456, (I) 314, (J) 355, (K) 306, (L) 337, (M) 300, (N) 623, (O) 119, (P) 152, (Q) 159, (R) 139, (S) 166, (T) 256 and (U) 264 aligned nucleotide positions using the GTRCAT model. Maximum likelihood bootstrap percentages and Bayesian Inference posterior probabilities are shown above and below branches respectively. Terminal nodes are labelled with the flanking DNA of the insert. The scale bar represents the number of substitutions per site. The phylogenies are formatted in the style of Figure 4.
